# A new murine gram-negative sepsis model with standard care satisfies Sepsis-3 and reproduces clinical pathology

**DOI:** 10.1186/s40635-026-00886-5

**Published:** 2026-03-30

**Authors:** Cameron R. Bastow, Cynthia Mei, Shu Wen Wen, Jenny L. Wilson, Huynh Nguyen, Althea R. Suthya, Joshua H. Bourne, Yugeesh R. Lankadeva, Connie H. Y. Wong

**Affiliations:** 1 Centre for Inflammatory Diseases, Department of Medicine, School of Clinical Sciences at Monash Health, Monash University, Clayton, VIC Australia; 2 Translational Cardiovascular and Renal Research Group, Florey Institute of Neuroscience and Mental Health, The University of Melbourne, Melbourne, VIC Australia; 3 Department of Critical Care, Melbourne Medical School, The University of Melbourne, Melbourne, VIC Australia; 4 Department of Anaesthesia, Austin Hospital, Melbourne, VIC Australia

## Abstract

**Background:**

Sepsis accounts for approximately a third of global mortality, and significant morbidity and economic burden. Whilst the current Sepsis-3 definition has augmented patient identification, supportive care and survival, a lack of clinically relevant animal models has limited our understanding of sepsis disease dynamics over time. Specifically, key knowledge gaps in chronic pathology underpinning the mechanisms leading to organ dysfunction and mortality rates of sepsis survivors have hindered the development of effective therapeutics. Therefore, we developed a new mouse model of abdominal gram-negative sepsis that adheres to Sepsis-3 definitions and expert-led consensus criteria for preclinical sepsis models.

**Results:**

We tested multiple live strains of *Escherichia coli* with only clinical isolates causing lethality. Subsequent standard care including broad-spectrum antibiotics and fluid resuscitation reduced the mortality rate to approximately 24 ± 9.3% (SEM), corroborating clinical observations. Early sepsis disease 12 h post-infection was characterized by cytokine storm, with concentrations of IFN-γ, CCL2, IL-6, IL-17A, IL-1α, IL-10 and M-CSF significantly elevated in multiple tissues up to 7 days post-infection when mice had recovered from objective clinical measures of disease. Furthermore, we observed histological evidence of organ dysfunction in the liver, spleen and kidney at 12 h to 3 days post-infection, validating concurrently increased serum markers of organ damage in our model. Additionally, infected mice treated with standard care exhibited persistent haematological dysfunction, as evidenced by anaemia, thrombocytosis and neutrophilia, at recovery from organ dysfunction 7 days post-infection, features similarly observed in clinical sepsis patients.

**Conclusions:**

Our new abdominal gram-negative murine sepsis model recapitulates key disease outcomes observed in sepsis patients and allows the study of dysfunctional homeostasis in surviving animals. This model can be utilized to identify and test new therapeutics for abdominal gram-negative sepsis or investigate novel mechanisms of immune dysfunction in sepsis survivors. Modifications to our murine model by utilizing alternate clinical pathogens, routes of infection, and mixed-sex, outbred or aged mice are necessary to recapitulate clinical sepsis heterogeneity and address the inherent limitations of preclinical models. Here, our methodology to establish a model with clinical isolates, satisfaction of Sepsis-3 definitions and preclinical sepsis guidelines provides a framework for the development of future models.

**Supplementary Information:**

The online version contains supplementary material available at 10.1186/s40635-026-00886-5.

## Background

Sepsis is a global health crisis responsible for approximately 32% of deaths, and it is accountable for significant economic burden worldwide [1–3]. Despite the prevalence of sepsis, current treatments for patients remain largely supportive in nature. Here, an incomplete understanding of the mechanisms underlying sepsis pathology and organ dysfunction has limited the development of new therapeutics. This is compounded by the poor translation of putative therapeutics identified in preclinical models which lack standardized guidelines unlike other disease models [4, 5]. Furthermore, preclinical sepsis models have remained relatively unchanged for decades despite the evolving clinical definition of sepsis. The current third iteration, denoted Sepsis-3, defines sepsis as life-threatening organ dysfunction caused by a dysregulated host response to infection [6].

To this end, the Minimum Quality Threshold in Preclinical Sepsis Studies (MQTiPSS) was established in 2018 to guide the development of preclinical models that satisfy the evolving definition of sepsis [7–10]. Central to these guidelines is the importance of recapitulating clinical pathogens, infection routes, interventions and timelines in animal models of disease. Notably, the majority of clinical sepsis cases are caused by monomicrobial infections with pathogenic strains of *Escherichia coli* (*E. coli*), *Staphylococcus aureus* (*S. aureus*), *Pseudomonas aeruginosa* (*P. aeruginosa*), *Klebsiella pneumoniae* (*K. pneumoniae*), or *Streptococcus pneumoniae* (*S. pneumoniae*) [3, 11–13]. Whilst the clinical prevalence of these species varies based on patient geographical location and the tissue origin of infection, gram-negative *E. coli* infections originating from urogenital and abdominal sources are the most common [3, 11–13]. Accordingly, new ovine models of *E. coli* sepsis that utilize clinical bacterial isolates have been developed to closely model sepsis cardiopulmonary pathophysiology and identify novel therapeutics [14, 15]. However, ovine models are not without their limitations. These include their high costs, technical specialty, low throughput, lack of commercially available antibodies for performing molecular investigations, restrictions in investigating sex differences as studies are limited to female ewes and castrated males, and logistical difficulties in performing aged studies.

Considering the limitations of large-animal models, the field has utilized rodent models to study sepsis pathophysiology, most commonly inoculation of animals with bacterial toxins such as lipopolysaccharide (LPS) or the caecal ligation puncture (CLP) model whereby a laparotomy facilitates breaching of the cecum and leaking of faecal microbiota into the abdomen to establish polymicrobial infection. In combination with insights gained from sepsis patients, human in vivo endotoxin studies and large‑animal models, traditional rodent models have substantially informed our understanding of sepsis pathology, yet they fail to capture several critical clinical features outlined in the MQTiPSS. Furthermore, the majority of the current models investigate early experimental endpoints which provide insight into acute sepsis cytokine storm, but largely ignore the long-term effects of surviving sepsis that are characterized by a striking increased risk of recurrent infections and death compared to survivors of non-infectious critical illness [16–18]. As such, there is an urgency in developing new preclinical models that recapitulate clinical features of the disease to improve development of effective therapies. To address the shortcomings of preclinical models, we generated a mouse model of abdominal sepsis with a primary clinical *E. coli* isolate. By implementing standard care encompassing broad-spectrum antibiotics and fluid resuscitation when infected mice meet Sepsis-3 descriptors, our new model satisfies MQTiPSS criteria, permits the study of both the early and late stages of sepsis, and can be utilized to identify and test new therapeutics in the context of clinical standard care.

## Results

### Pathogenic clinical *E. coli* isolates trigger fatal multi-organ dysfunction in mice

**Fig. 1:**
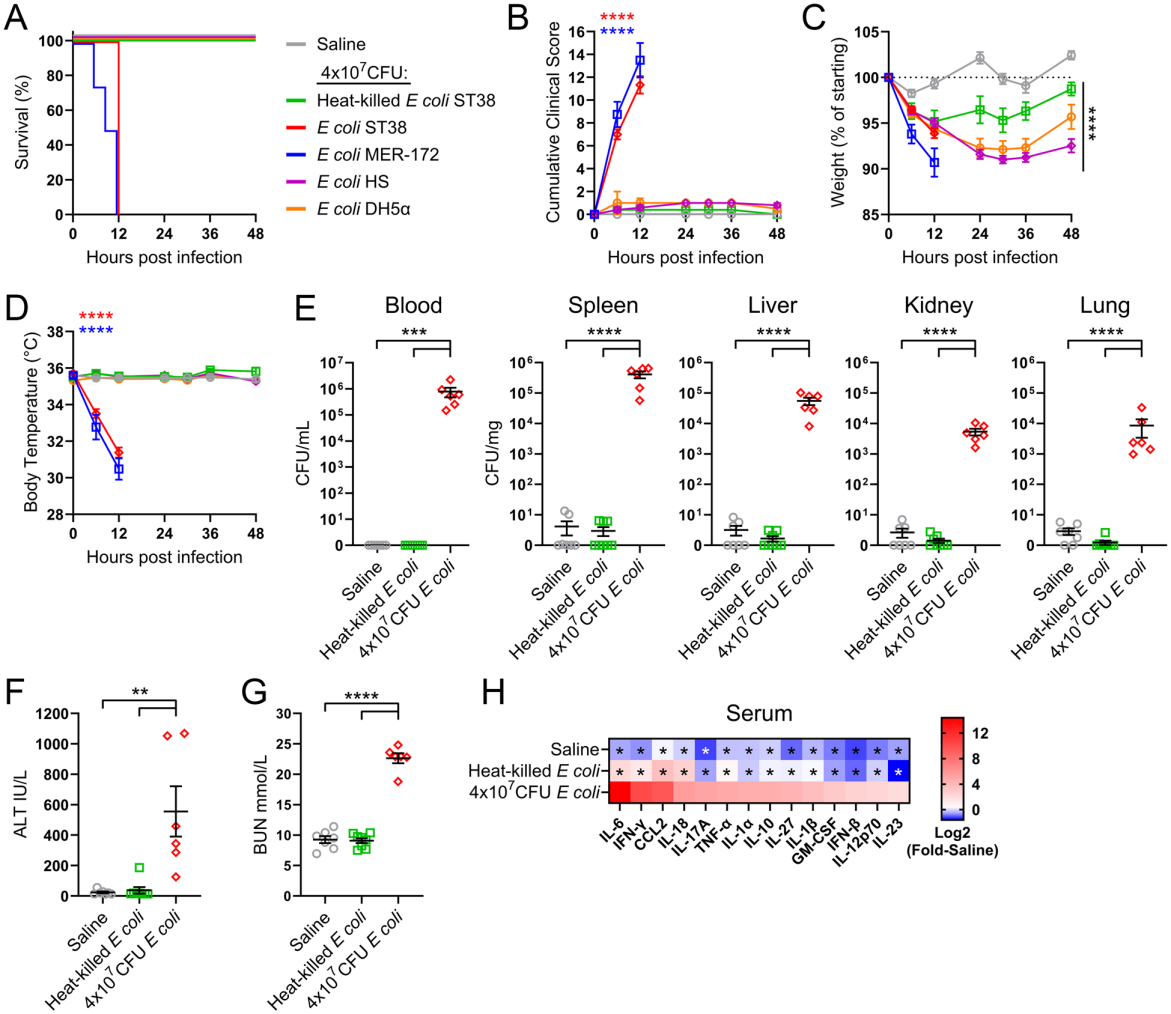
Clinical *E. coli* strains drive fatal abdominal sepsis in mice. Mice were infected intraperitoneally (i.p.) with 4×10^7^ CFU of *E. coli* strains of varying origin and **A** survival, **B** cumulative clinical score, **C** weight loss and **D** body temperature were measured over 48 h. Data pooled from 3 independent experiments, saline n=5, heat-killed *E. coli* n=5, *E. coli* ST38 n=6, *E. coli* MER-172 n=4, *E. coli* HS n=5, *E. coli* DH5α n=4 mice. **B–D** Two-way main effects ANOVA with Dunnett’s multiple comparisons test, displayed as mean ± SEM with statistical comparisons to saline control. Infected mice were assessed for **E** bacterial burden in various organs, **F** alanine transaminase (ALT), **G** blood urea nitrogen (BUN) and **H** serum inflammatory cytokines at 12 h post-infection. **E–G** Data pooled from 2 independent experiments, saline n=7, heat-killed *E. coli* n=8, *E. coli* ST38 n=6 mice, each symbol is one mouse with mean ± SEM. **E** Lognormal ordinary ANOVA with Tukey’s multiple comparisons test, except blood, Kruskal–Wallis test with Dunn’s multiple comparison test, **F** Kruskal–Wallis test with Dunn’s multiple comparison test, **G** one-way ANOVA with Tukey’s multiple comparisons test. **H** Data are log2-fold increase above saline and pooled from 3 independent experiments, saline n=7 (except for IL-18, n=4), heat-killed *E. coli* n=8, *E. coli* ST38 n=6 mice/analyte, two-way simple effects ANOVA, groups compared to 4×10^7^ CFU *E. coli* with Dunnett’s multiple comparison test. *p < 0.05, **p < 0.01, ***p < 0.001, ****p < 0.0001.

To generate a clinically relevant mouse model of sepsis, we sourced a clinical *E. coli* strain previously isolated from a sepsis patient and utilized in ovine models [14]. Sequencing identified this isolate as sequence type 38 (ST38), serotype O45:H14 and phylotype D (hereafter *E. coli* ST38, Supplemental Fig. 1A). Isolates within the ST38 lineage are within the top 5 most prevalent strains found in extraintestinal pathogenic *E. coli* (ExPEC) infections globally [19, 20]. Identification of virulence factor genes implicated *E. coli* ST38 as a hybrid intestinal pathogenic *E. coli* (IPEC)/ExPEC pathotype as reported for other ST38 isolates [21] (Supplemental Fig. 1A, B; Supplemental Table 1), and antimicrobial resistance genes revealed putative resistance to aminoglycosides and β-lactams commonly used to treat *E. coli* infections (Supplemental Table 2, Supplemental Fig. 1C). The ST38 isolate shared multiple virulence factors with another sepsis isolate from the prevalent ST131 lineage, *E. coli* strain MER-172 [22] (Supplemental Fig. 1A, Supplemental Table 1). Comparatively, a non-pathogenic human commensal *E. coli* HS strain [23] possessed fewer virulence genes and lacked antimicrobial resistance genes (Supplemental Fig. 1, Supplemental Table 1). To establish our abdominal sepsis model, we identified the lowest infectious dose of *E. coli* ST38 required for 100% lethality in adult mice, as the resulting multi-organ dysfunction would be “life-threatening” and untreated clinical sepsis is fatal. Here, 4×10^7^ colony forming units (CFU) of *E. coli* ST38 and MER-172 resulted in all mice reaching humane euthanasia endpoints by 12 h post-infection (Fig. 1A). Half the lethal dose of *E. coli* ST38 (2×10^7^ CFU) displayed 30% lethality at 30 h post-infection with 5/7 infected mice tolerating this dose and recovering without intervention (Supplemental Fig. 2A-D). Importantly, lethality was not a result of immediate endotoxic shock as all mice survived an equivalent heat-killed dose of *E. coli* ST38 (Fig. 1A). Only pathogenic *E. coli* isolates established lethal infection at this concentration, as the commensal *E. coli* HS strain and laboratory DH5α strain did not impact survival (Fig. 1A). Lethal *E. coli* ST38 and MER-172 infected mice displayed significant clinical signs of illness as measured by reduced activity, alertness, respiratory depression and dehydration (Fig. 1B), weight loss (Fig. 1C) and hypothermia (Fig. 1D). Weight loss was also observed in mice infected with heat-killed *E. coli* ST38 or non-pathogenic *E. coli* strains (Fig. 1B–D). Given the prevalence of uropathogenic *E. coli*-associated (UPEC) virulence factors expressed by the MER-172 strain (Supplementary Table 1), we proceeded to develop our abdominal gram-negative sepsis model with the hybrid IPEC/ExPEC ST38 *E. coli* strain as this best recapitulated its natural route of infection.

At the peak of disease 12 h post-infection with *E. coli* ST38, significant bacterial burden was observed in the blood and exsanguinated major organs including the spleen, liver, kidney and lungs (Fig. 1E). Concurrently, alanine transaminase (ALT) and blood urea nitrogen (BUN) levels were significantly elevated in the blood (Fig. 1F, G), indicative of liver and kidney dysfunction, respectively. Significantly increased concentrations of cytokines characteristic of a systemic inflammatory response, including TNF-α, IL-1β, IL-18, IL-6, IFN-γ, IL-17, and IL-10 [24, 25], were detected in the serum of mice infected with viable *E. coli* ST38 (Fig. 1H, Supplemental Fig. 3A-N). Collectively, the clinical *E. coli* ST38 isolate caused fatal disease in mice characterized by disseminating bacteraemia and organ dysfunction, fulfilling the Sepsis-3 descriptors of sepsis [6].

### Standard clinical care rescues septic mice

**Fig. 2:**
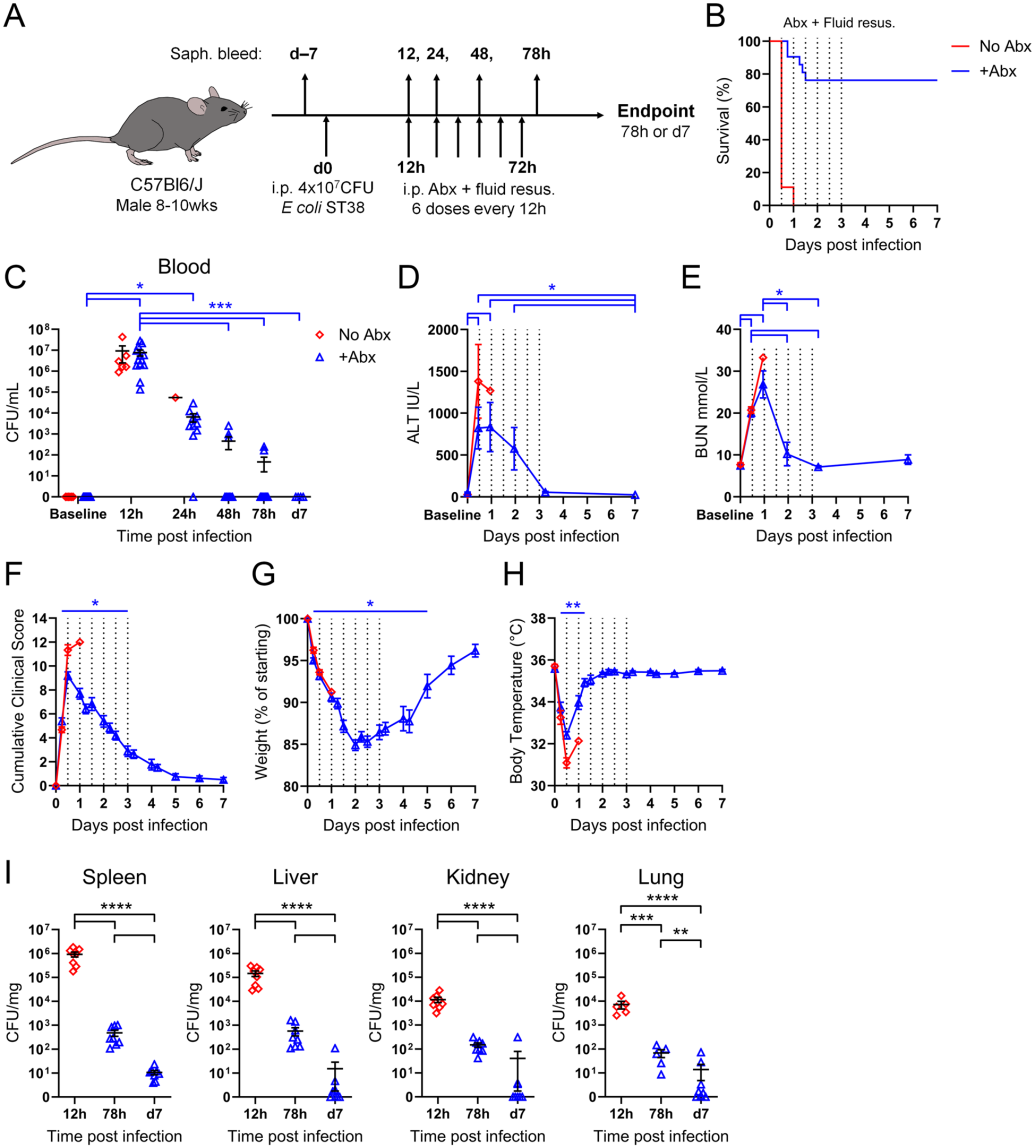
Initiating standard care upon fulfilling Sepsis-3 criteria augments mouse survival and recovery. **A** Schematic timeline of standard care regime. Mice were infected i.p. with 4×10^7^ CFU of *E. coli* ST38, then treated i.p. with 6 doses of antibiotics and saline fluid resuscitation at 12 h intervals starting 12 h post-infection. Blood samples were collected from the saphenous vein at indicated experimental timepoints. **B** Survival of septic mice with and without standard clinical care, n=21 and 9, respectively. Data pooled from 6 independent experiments. **C** Blood bacterial burden, **D** ALT and **E** BUN were measured at baseline, before initiation of standard care regime and during recovery. **C, D, E** Data pooled from 3 independent experiments, no antibiotics n=6, antibiotics n=13, Kruskal–Wallis ANOVA with Dunn’s multiple comparisons test on antibiotic-treated animals. **C** Each symbol is one mouse with mean ± SEM, **D, E** displayed as mean ± SEM. **F** Cumulative clinical score, **G** weight loss and **H** body temperature were measured over 7 days, and **I** bacterial burden in organs was enumerated at experimental endpoints. **F–H** Data pooled from 6 independent experiments, no antibiotics n=9, antibiotics n=21 mice, displayed as mean ± SEM. Kruskal–Wallis ANOVA with Dunn’s multiple comparisons test on antibiotic-treated animals compared to baseline. **I** Data pooled from 6 independent experiments, n=8 mice/timepoint, except lung where 12 h n=5, 78 h n=5, d7 n=8 mice. Each symbol is one mouse with mean ± SEM. Lognormal ordinary ANOVA with Tukey’s multiple comparisons test. *p < 0.05, **p < 0.01, ***p < 0.001, ****p < 0.0001.

In clinical settings, immediate broad-spectrum antibiotic treatment and fluid resuscitation are standard care for patients with suspected sepsis. Delayed antibiotic treatment increases mortality in both patients and preclinical models of sepsis [26–28]. For this reason, multiple iterations of the Surviving Sepsis Campaign have described guidelines to augment the identification and early treatment of sepsis patients, reducing sepsis mortality rates to 15–25% within high-income countries [3, 11, 13, 29]. To recapitulate the clinical scenario where standard care is given after evidence of septic symptoms, we initiated prolonged broad-spectrum carbapenem antibiotic treatment and crystalloid fluid resuscitation (hereby “standard care”) at 12 h post-infection (Fig. 2A), a timepoint where all mice fulfilled Sepsis-3 criteria (Fig. 1). Notably, the clinically relevant standard care regime of continued antibiotic treatment and fluid resuscitation permitted the recovery of septic mice, reducing mortality to 24 ± 9.3% (SEM) (Fig. 2B). Antibiotic treatment at 12 h post-infection significantly reduced bacteraemia burden within 36 h (Fig. 2C). Liver dysfunction, as measured by increased serum ALT, persisted over the 72 h regime of standard care, before the baseline ALT levels were restored between 3 and 7 days post-infection (Fig. 2D). Comparatively, kidney dysfunction as evidenced by elevated BUN levels, were raised within 12 h of infection but rapidly resolved to normal BUN levels within 2 days (Fig. 2E). Additionally, standard care facilitated the improvement of clinical illness symptoms relating to activity and alertness (Fig. 2F), restored sepsis-induced weight loss (Fig. 2G), and rapidly resolved disease-associated hypothermia (Fig. 2H). Antibiotic treatment cleared infection in the liver, kidney and lungs within 7 days, with seldom mice harbouring detectable levels of bacteria in these organs (Fig. 2I). However, detectable levels of bacteria were measured in the spleens of all mice 7 days post-infection (Fig. 2I). Thus, our preclinical model that implemented current standard care regimes replicated a best-case scenario of clinical treatment, whereby appropriate antibiotics are rapidly administered upon speculation of sepsis. By mirroring the same standard of clinical care in our preclinical model, we recapitulated a clinically relevant mortality rate of ~20% in an otherwise fatal infection.

### Sepsis induces diverse cytokine responses in various tissues

**Fig. 3:**
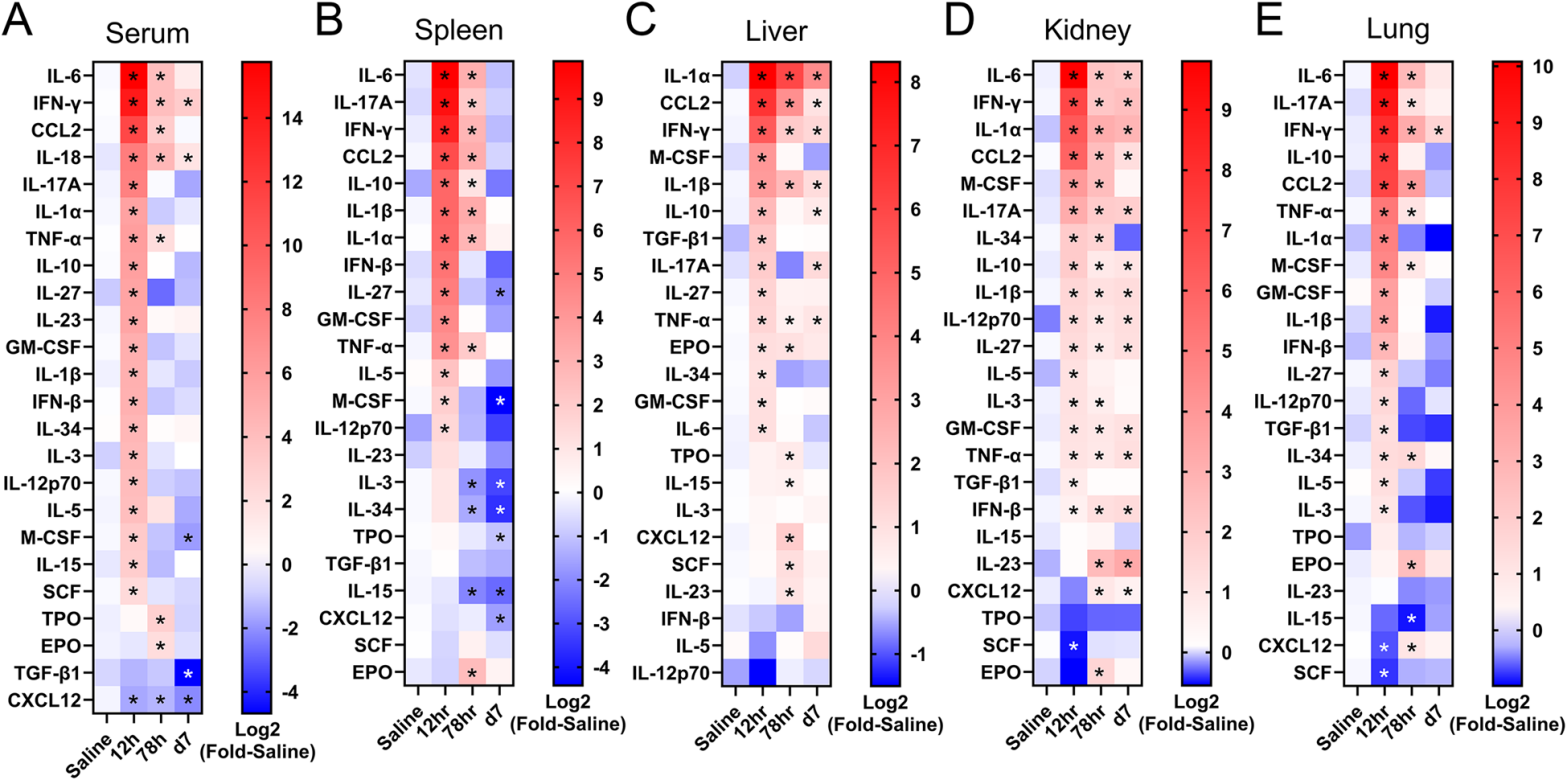
Inflammatory and haematopoietic cytokines are elevated in the blood and organs of septic mice throughout recovery. Inflammatory and haematopoietic cytokines were measured in the **A** serum, and homogenates of the **B** spleen, **C** liver, **D** kidney and **E** lung of saline-treated mice or mice at 12 h, 78 h and 7 days post-infection with 4×10^7^ CFU *E. coli* ST38 followed by standard care. **A** Data are log2-fold increase above saline and pooled from 5 independent experiments, saline n=6–7, 12 h n=3–4, 78 h n=5 (except for IL-18, n=8), d7 n=4 mice/analyte (also see Supplemental Fig. 4), ordinary one-way ANOVA with Dunnett’s multiple comparison test compared to saline control for each cytokine. **B–E** Data are log2-fold increase above saline and pooled from 5 independent experiments, saline n=7, 12 h n=6, 78 h n=5, d7 n=4 mice/analyte, ordinary one-way ANOVA with Dunnett’s multiple comparison test compared to saline control for each cytokine. *p < 0.05.

To understand the immunological signalling that underpins the systemic dysfunction in septic mice, extensive inflammatory and haematopoietic cytokine profiling was performed on the blood and exsanguinated organ homogenates. Consistent with the well-documented sepsis-induced acute cytokine storm, most cytokines assayed were elevated in the serum at the peak of disease 12 h post-infection (Fig. 3A, Supplemental Fig. 4A-X). In particular, we observed more than tenfold increase in the levels of pro-inflammatory IL-6, IFN-γ, CCL2, IL-18, IL-17A, TNF-α and anti-inflammatory IL-10 compared to saline-treated healthy control counterparts (Fig. 3A). Concentrations of IL-6, IFN-γ, CCL2, IL-18 and TNF-α remained greater than healthy controls at the conclusion of standard care 78 h post-infection (Supplementary Fig. 4A-D, G). Similarly, most haematopoietic cytokines were increased in concentration at 12 h post-infection; however, TPO and EPO levels in the sera were greatest at the onset of recovery at 78 h post-infection (Fig. 3A, Supplementary Fig. 4U, V). Conversely, CXCL12 abundance was significantly diminished after infection (Fig. 3A, Supplementary Fig. 4X).

Similar cytokine kinetics were observed in the spleen (Fig. 3B, Supplemental Fig. 5A–W). Concentrations of IL-6, IL-17A, IFN-γ, CCL2, IL-10, IL-1β, IL-1α, IFN-β, IL-27, GM-CSF and TNF-α were all significantly elevated by greater than tenfold during peak disease (Supplemental Fig. 5A-K). Whilst the levels of most of these cytokines rapidly resolved, IL-6, IFN-γ, CCL2, IL-1β and IL-1α remained significantly elevated at 78 h post-infection compared to control (Supplemental Fig. 5A, C, D, F, G). As observed in the blood, EPO levels transiently peaked at the onset of recovery at 78 h post-infection (Supplemental Fig. 5W). Interestingly, multiple haematopoietic cytokines at 7 days post-infection were diminished below concentrations observed in uninfected mice, including M-CSF, IL-34, IL-15 and the chemokine CXCL12 (Supplemental Fig. 5M, Q, T, U).

The liver was the only tissue assayed where IL-6 was not the most upregulated cytokine (Fig. 3C, Supplemental Fig. 6A-W). Here, IL-1α and IFN-γ abundance was significantly upregulated at 12 h post-infection, and despite decreasing over the following 7 days, the levels of sepsis-induced hepatic IL-1α and IFN-γ remained significantly elevated compared to healthy levels (Fig. 3C, Supplemental Fig. 6A, C). Similarly, the abundance of CCL2, M-CSF, IL-1β, IL-10, IL-27, TNF-α, and IL-34 were significantly increased in the liver at peak disease before resolving over the course of recovery (Supplemental Fig. 6B, D-F, I, J, L). The concentrations of CXCL12 and SCF significantly increased by 78 h post-infection, with similar dynamics observed for IL-23 in some mice (Supplemental Fig. 6R-T).

Contrary to other organs, multiple cytokines demonstrated sustained upregulation in the kidney up to 7 days post-infection (Fig. 3D, Supplemental Fig. 7A-W), including IL-6, IFN-γ, IL-1α, CCL2, IL-17A, IL-10, IL-1β, IL-12p70, IL-27, GM-CSF, TNF-α and IFN-β (Supplemental Fig. 7A-D, E, H–K, N, O, Q). Interestingly, the renal concentrations of IL-23, CXCL12 and EPO showed a delayed upregulation after disease onset, with elevated levels detected from 78 h post-infection (Supplemental Fig. 7S, T, W). 

Lastly, cytokine kinetics in the septic lung largely mirrored those observed in the serum and spleen (Fig. 3E, Supplemental Fig. 8A-W). Proinflammatory cytokines IL-6, IL-17A, IFN-γ, CCL2, TNF-α and IL-1α were significantly increased by an order of magnitude at peak disease (Supplementary Fig. 8A-C, E–G), with only IFN-γ concentrations remaining above healthy baseline 7 days post-infection. Similarly, anti-inflammatory IL-10 and haematopoietic M-CSF concentrations were increased greater than tenfold during peak disease (Supplementary Fig. 8D, H). At the conclusion of standard care and onset of recovery 78 h post-infection, IL-34, EPO and CXCL12 were increased in the lung (Supplementary Fig. 8O, S, V).

Collectively, IFN-γ and CCL2 were consistently within the top significantly increased cytokines across all tissues analysed, whilst IL-6, IL-17A, IL-1α, IL-10 and M-CSF were significantly elevated in multiple tissues. Generally, cytokine concentrations peaked at 12 h and resolved within 78 h post-infection, with the kidney being the slowest to reduce cytokine levels. Interestingly, a transient increase in EPO was observed within all tissues by 78 h, and multiple haematopoietic cytokines were diminished below steady-state levels in the spleen after 7 days.

### Kinetics of multi-organ dysfunction correlate with histological tissue damage

**Fig. 4:**
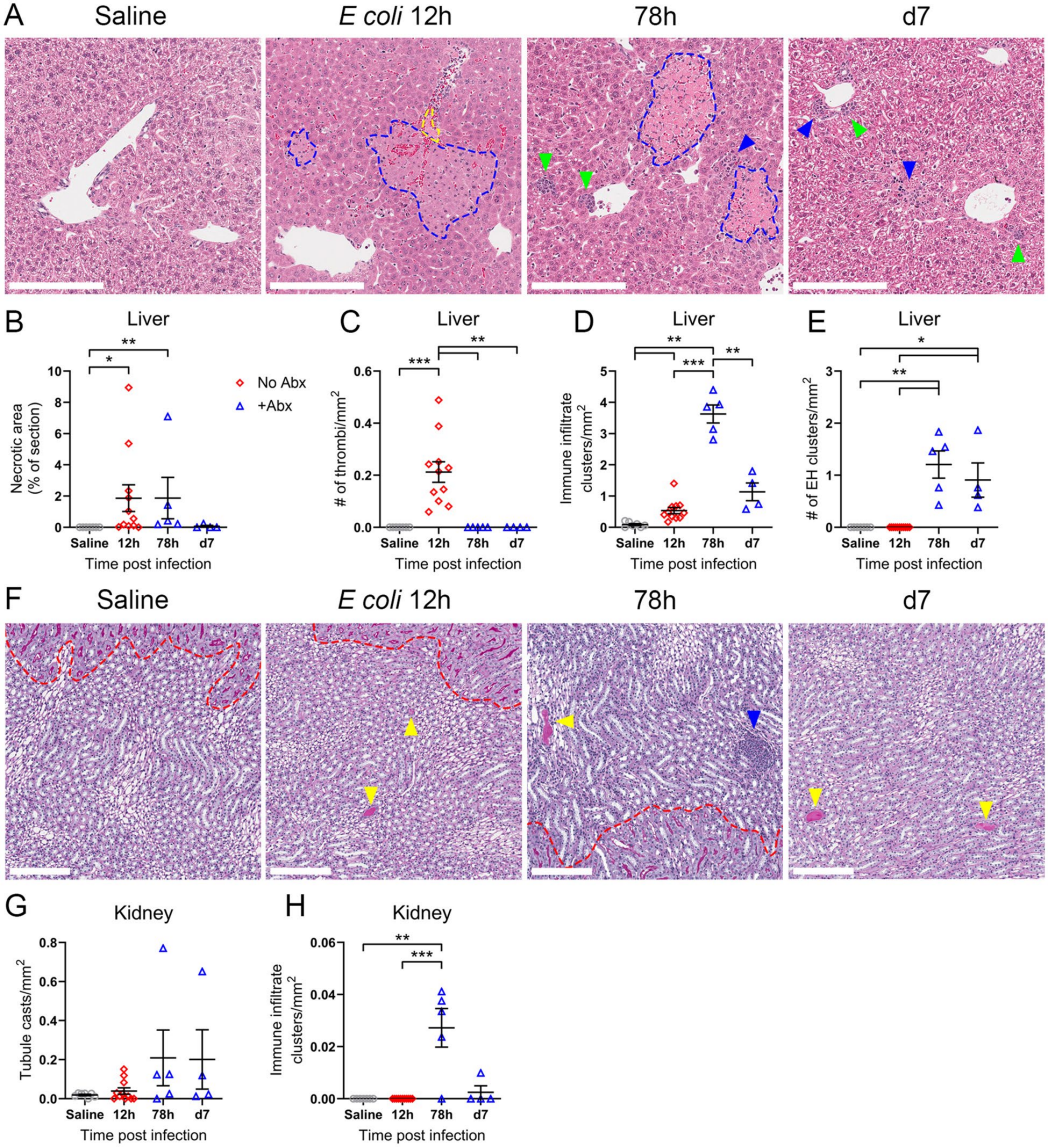
Hepatic and renal dysfunction is evident by histological analyses. **A** Representative haematoxylin and eosin (H&E) stained liver sections from control saline mice and mice at 12 h, 78 h and 7 days post-infection with 4×10^7^ CFU *E. coli* ST38 followed by standard care. Scale bar = 200 μm, blue dotted outline denotes focal necrosis, yellow dotted outline denotes thrombi, blue arrowheads highlight immune infiltrate clusters and green arrowheads delineate extramedullary haematopoiesis (EH) clusters. Enumerated **B** necrosis, **C** thrombi, **D** immune infiltrate clusters and **E** EH clusters in the liver from **A**. **F** Representative periodic acid-Schiff (PAS) stained kidney medulla sections from control saline mice and mice at 12 h, 78 h and 7 days post-infection with 4×10^7^ CFU *E. coli* ST38 followed by standard care. Scale bar = 200 μm, red dotted line demarcates the corticomedullary junction, yellow arrowheads highlight tubule casts and blue arrowheads highlight immune infiltrate clusters. Enumerated **G** tubule casts and **H** immune infiltrate clusters in the kidney from **F**. **A–H** Images representative of pooled data from 5 independent experiments, saline n=7, 12 h n=11, 78 h n=5, d7 n=4 mice. **B–E, G, H** Each symbol is one mouse with mean ± SEM. **B** Brown–Forsythe and Welch ANOVA with Dunnett’s T3 multiple comparisons test, **C, D, E, G, H** Kruskal–Wallis ANOVA with Dunn’s multiple comparisons test. *p < 0.05, **p < 0.01, ***p < 0.001.

To further validate the elevated serum markers of organ damage present in our model, we examined the liver and kidneys of septic mice over time by histology. Healthy liver tissue in saline-treated mice was exemplified by extensive glycogen accumulation (clear vacuoles) in hepatocytes (Fig. 4A). At peak disease, glycogen accumulation was lost and focal hepatocyte necrosis was observed in pericapsular and centrilobular areas (Fig. 4A, blue outline), the latter of which was often observed in proximity to occluding thrombi (yellow outline). Hepatic necrotic lesions persisted until 78 h post-infection (Fig. 4A, B), whilst thrombi were rapidly cleared (Fig. 4C). Immune infiltrates (Fig. 4A, blue arrowheads) emerged as early as 12 h post-infection, but were most prominent at the onset of recovery before declining by day 7 (Fig. 4D). During recovery, cell clusters indicative of extramedullary haematopoiesis (EH) were common in perivascular areas [30, 31] (Fig. 4A, green arrowheads, Fig. 4E) and hepatocyte glycogen accumulation was restored by day 7. Thus, our model replicated focal hepatocyte necrosis as observed in clinical sepsis [32], and this was concurrent with elevated circulatory ALT present throughout standard care.

Sepsis is a leading cause of acute kidney injury (S-AKI) and there is a significant association between kidney dysfunction and sepsis mortality [33]. The factors underlying S-AKI are incompletely understood; however, previous studies have attributed prolonged exposure of renal cells to inflammatory cytokines as a contributing factor [33], as we also observe (Fig. 3D, Supplementary Fig. 7), and reported a lack of extensive renal cell death in post-mortem biopsies [32, 34]. Similarly, histological evidence of kidney damage was scarce in our model, but when present, was largely limited to the renal medulla and areas adjacent to the corticomedullary border (Fig. 4F, red dotted line). As sepsis progressed, there was a trend towards increased tubule protein cast formation (Fig. 4F, yellow arrowheads); however, this did not reach statistical significance (Fig. 4G). This periodic acid Schiff (PAS)-positive tubule cast staining was characteristic of hyaline casts, of which the predominate constituent, uromodulin, has described roles in tubular cell protection against *E*.* coli* [35, 36]*.* Medullary immune infiltrates were observed at 78 h post-infection (Fig. 4F, blue arrowhead) but absent at other timepoints (Fig. 4H). Thus, the limited gross renal tissue damage observed in our preclinical model is in line with previous sepsis studies and implicates alternate mechanisms such as metabolic and haemodynamic dysfunction in S-AKI [33, 37].

### Extensive haematopoietic dysfunction persists in mice that survive sepsis

**Fig. 5:**
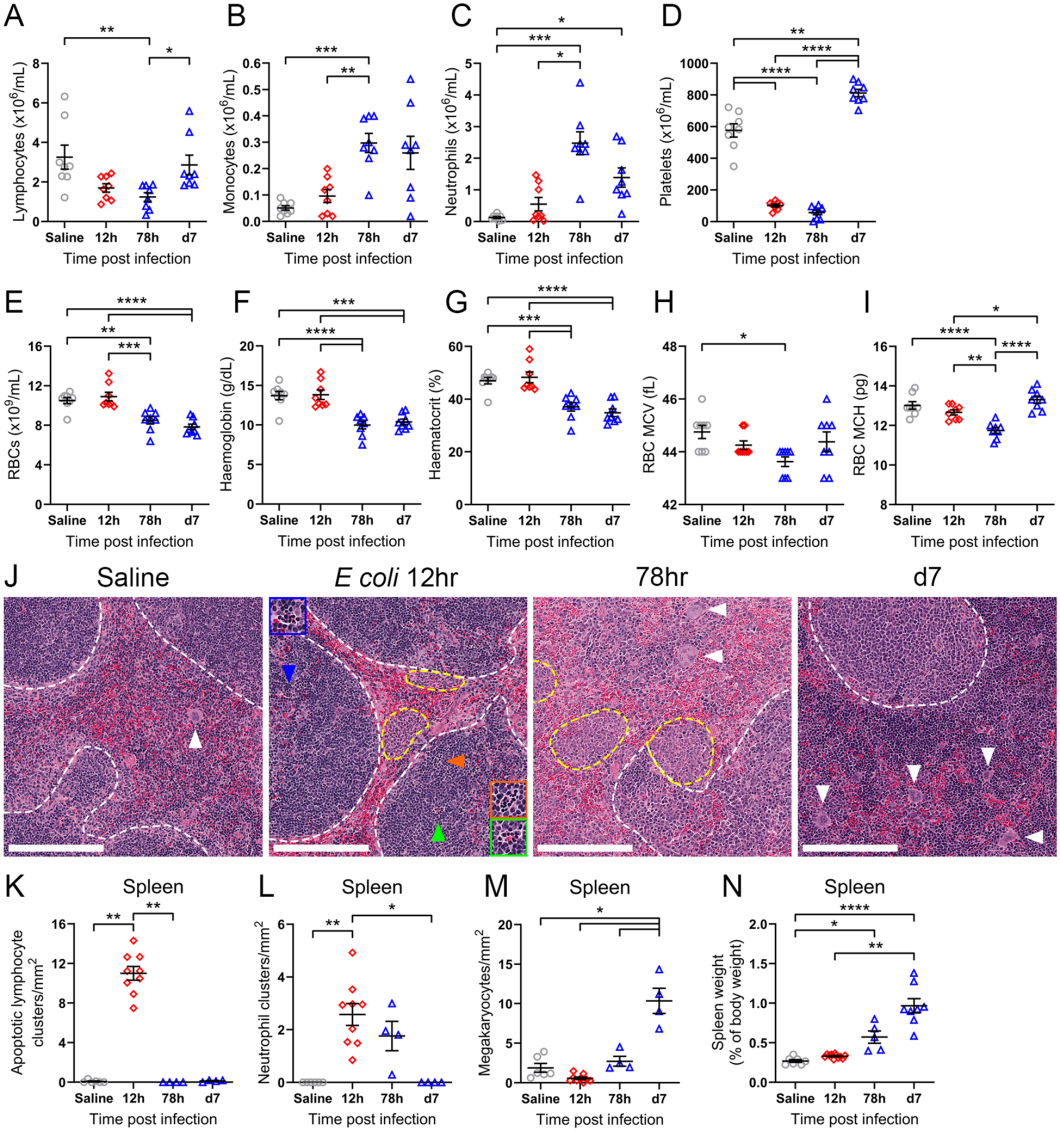
Haematopoietic and splenic abnormalities persist in sepsis-recovered mice. Blood was harvested from control saline mice and mice at 12 h, 78 h and 7 days post-infection with 4×10^7^ CFU *E. coli* ST38 followed by standard care. Subsequently the abundance of circulatory **A** lymphocytes, **B** monocytes, **C** neutrophils, **D** platelets and **E** red blood cells (RBC) were measured, and **F** haemoglobin, **G** haematocrit, **H** RBC mean corpuscular volume (MCV), and **I** RBC mean corpuscular haemoglobin (MCH) was quantified. **A–I** Data pooled from 7 independent experiments, n=8 mice/timepoint, each symbol is one mouse with mean ± SEM. **A, C** Kruskal–Wallis ANOVA with Dunn’s multiple comparisons test, **B, D** Brown–Forsythe and Welch ANOVA with Dunnett’s T3 multiple comparisons test, **E–I** ordinary one-way ANOVA with Tukey’s multiple comparisons test. **J** Representative H&E stained spleen sections from control saline mice and mice at 12 h, 78 h and 7 days post-infection with 4×10^7^ CFU *E. coli* ST38 followed by standard care. Scale bar = 200 μm, white dotted outline demarcates the white pulp, yellow dotted outlines denote neutrophil clusters, white arrowheads highlight megakaryocytes and coloured arrowheads delineate apoptotic lymphocyte clusters within zoomed panels. Enumerated **K** apoptotic lymphocyte clusters, **L** neutrophil clusters and **M** megakaryocytes in the spleen from **J**. Images representative of pooled data from 5 independent experiments, saline n=6, 12 h n=9, 78 h n=4, d7 n=4 mice. **K, L, I** Each symbol is one mouse with mean ± SEM. **K, L** Kruskal–Wallis ANOVA with Dunn’s multiple comparisons test, **M** Brown–Forsythe and Welch ANOVA with Dunnett’s T3 multiple comparisons test. **N** Spleen weights pooled from 7 independent experiments, n=5-11 mice/timepoint, each symbol is one mouse with mean ± SEM, Kruskal–Wallis ANOVA with Dunn’s multiple comparisons test. *p < 0.05, **p < 0.01, ***p < 0.001, ****p < 0.0001.

Lymphopenia is observed in sepsis patients and contributes to immunosuppression [38]. Similarly, we observed a decrease in circulating lymphocytes in septic mice (Fig. 5A), and this coincided with increased numbers of monocytes (Fig. 5B) and neutrophils (Fig. 5C). Whilst sepsis recovery 7 days post-infection was associated with a rebound in lymphocyte numbers, neutrophilia persisted (Fig. 5A, C). As observed in sepsis patients [39–41], thrombocytopenia rapidly developed within 12 h of infection and persisted up to 78 h post-infection in mice (Fig. 5D). However, 7 days after infection, we found that mice had platelet counts significantly higher than healthy control animals (Fig. 5D). This phenomenon was indicative of secondary thrombocytosis as described in recovering sepsis patients [39–41]. Additionally, prolonged inflammation in septic patients drives anaemia of inflammation (also known as anaemia of chronic disease) via decreasing iron transport, EPO signalling and red blood cell (RBC) survival [42–44]. In line with these clinical characteristics, we observed significant reductions in RBC abundance, blood haemoglobin concentration and haematocrit in septic mice at both 78 h and 7 days post-infection (Fig. 5E–G). Furthermore, the mean corpuscular volume (MCV), a measure of average RBC size, and mean corpuscular haemoglobin (MCH), the average amount of haemoglobin per RBC, were significantly reduced at 78 h post-infection (Fig. 5G, H). Here, reduced MCV and MCH were indicative of microcytic and hypochromic anaemia at 78 h post-infection [45]. Whilst RBC size and haemoglobin content were restored to baseline levels by day 7, anaemia persisted given the reduced number of RBCs and total haemoglobin. Together, these measures demonstrated that septic mice established prolonged anaemia despite increased EPO expression (Figs. 3, 5E-G), suggesting that the factors driving anaemia during sepsis are complex and dynamic.

To further investigate haematological dysfunction in our septic mice, we examined immune cell composition in the spleen over the sepsis time-course by histology (Fig. 5J). The spleens of healthy mice contained clearly demarcated white and red pulp areas (Fig. 5J, white dotted lines) and few megakaryocytes within the red pulp (white arrowheads). As observed in sepsis patients post-mortem [32], peak disease in our preclinical model was marked by extensive lymphocyte apoptosis in the white pulp (Fig. 5J, coloured arrowheads and inserts) which resolved following standard care treatment (Fig. 5K). Clusters of neutrophils within proximity of the white pulp (Fig. 5J, yellow outline) were observed at both 12 and 78 h after infection (Fig. 5L), and resolved by 7 days. A significant increase in the number of megakaryocytes was evident at 7 days post-infection (Fig. 5J, white arrowheads), surpassing baseline levels (Fig. 5M). Indeed, increased splenic cellularity at 7 days post-infection resulted in disrupted zonal spleen architecture and significant splenomegaly (Fig. 5N). Together, these observations provide evidence that our preclinical model closely recapitulates the extensive and ongoing haematological dysfunction present in sepsis patients and survivors.

## Discussion

Poor translation of putative therapeutic targets from animal models of sepsis into the clinic signals an urgent need for paradigm shifts in the design of preclinical sepsis studies. A review of the top 260 most-cited preclinical sepsis studies between 2003 and 2012 revealed that 44% utilized the CLP model, whilst 40% utilized the acute LPS model [9]. In this study, we establish a murine model of abdominal sepsis utilizing a live, clinical *E*.* coli* isolate and standard care administered over a clinically appropriate timeframe after animals fulfil the clinical descriptors of sepsis. In line with the recent MQTiPSS guidelines [7], our preclinical model satisfies all applicable recommendations (19/19) and 63% (5/8) of optional considerations (Supplementary Table 3). Notably, our new preclinical model is distinguished from past models as it implements the essential current standard care practices to emulate clinical mortality rates. This aspect of the model is critical for translation of preclinical findings and discoveries, as novel therapeutics must demonstrate efficacy and improved patient outcomes when combined with standard care. As such, our model is poised to test new therapeutic targets in the context of standard care, and provides an effective platform to develop putative therapeutics in future studies.

In addition, the findings of our study demonstrate that pathogenic strains of *E*.* coli* are essential to elicit life-threatening organ dysfunction in mice. Such clinical bacterial isolates can vary in their virulence factors and antimicrobial resistance profiles based on the tissue of isolation, patient demographics, and geographical location. Therefore, preliminary purification and genomic characterization of clinical isolates are necessary before their use in preclinical studies. Notably, the adoption of genetically varied clinical strains by the research community in preclinical models presents multiple benefits. Firstly, inoculation with known doses of live, characterized pathogens permits greater precision and reproducibility of infection, limiting a considerable source of variation observed in animal models utilizing caecal microbiota to establish infection. Furthermore, employing a vast array of clinical pathogens in preclinical models will address a key concern that human-adapted pathogens may display host-restricted virulence and thus behave differently in alternate animal hosts. Indeed, limited strains of clinical sepsis pathogens, including *E*.* coli*, *S*.* aureus*, and *S*.* pneumoniae*, display virulence in mice [46–51]. Additionally, important bacterial virulence factors implicated in evasion of the human immune response, such as choline-binding protein A and leukocidins, are not cross-reactive in mice [52, 53]. By utilizing a broad range of clinical isolates, important conserved similarities in the immune response against these pathogens will be evident, and experimental artefacts attributable to a lack of species cross-reactivity will be highlighted. Moreover, key clinical virulence factors and immune evasion mechanisms that drive sepsis pathology may be identified, thus aiding the development of new prognostic biomarkers and targeted therapeutics. Lastly, systematic analysis of preclinical models that utilize various clinical strains may reveal sepsis subtypes and heterogeneity attributable to specific bacterial species, strains, or virulence factors. Indeed, multiple groups have defined different human sepsis subphenotypes by clinical data or blood leukocyte transcriptomic profiling [54–58]. Whilst common sepsis-induced immunosuppressive and coagulopathy signatures have been observed through these endeavours, the clinical implementation of sepsis subtypes is yet to be appreciated. With the increasing appreciation of omics technology, exploring preclinical sepsis subtypes in the context of different infections may reveal new markers to better inform on appropriate antimicrobial selection.

We observed extensive cytopenia and elevated compensatory extramedullary haematopoiesis in our preclinical model during the early and late stages of sepsis, respectively. Interestingly, a recent study utilizing single-cell sequencing approaches identified a sub-phenotype of sepsis patients with poor outcomes and characterized by increased immature neutrophils resultant of emergency granulopoiesis [54]. Here, aberrant granulopoiesis was driven by STAT3 signalling downstream of cytokines including IL-6 and G-CSF, and resulted in the expansion of immature neutrophils with an immunosuppressive phenotype capable of suppressing adaptive T cell responses in vitro [54]. Similarly, in our preclinical sepsis model we observed significantly increased abundance of IL-6 family cytokines known to signal through STAT3, evidence of multi-organ extramedullary haematopoiesis and neutrophilia. Thus, further investigation is required to interrogate the functionality of rapidly expanded immune cells and whether their development outside the bone marrow in organs such as the spleen and liver affects their function. In the context of sepsis, normal functioning of rapidly expanded immune cells may be crucial to protect sepsis survivors from opportunistic secondary infections, particularly in the lungs. Alternatively, immature immune cells may act as a cellular reservoir and insufficiently clear the infection, possibly explaining our observation that *E. coli* persisted in the spleen up to 7 days post-infection despite the administration of antibiotics. Therefore, future studies utilizing our preclinical model may investigate the contribution of nascent immune cells developed during sepsis via extramedullary haematopoiesis on immune responses to secondary pneumonia established by intranasal infection.

Moreover, we observed significant and prolonged thrombocytopenia established within 12 h post-infection and persisting beyond 3 days post-infection. The resulting overcorrection in platelet abundance at 7 days post-infection, termed secondary thrombocytosis, was concurrent with splenomegaly and a striking expansion of splenic megakaryocytes. Our observations mirror those in a recent preclinical sepsis study where significant splenic megakaryocyte expansion, partially dependent on IL-3, produced a protective pool of immunomodulatory CD40L^hi^ platelets that accounted for >13% of circulating platelets [59]. In that study, megakaryocyte precursors were mobilized from the bone marrow via reduced local SCF and CXCL12 production, and increased plasma SCF [59]. Similarly, we observed elevated SCF in the blood during the initial cytokine storm at 12 h post-infection; however, the spleen was largely deficient in haematopoietic cytokines including IL-3 at late timepoints when megakaryocyte expansion was present. Given that IL-3 has been implicated in emergency myelopoiesis and cytokine storm within 24 h after CLP [60], it is likely that alternate cytokines promoted megakaryocyte differentiation in our model. Nonetheless, our preclinical observations align with biphasic platelet kinetics observed in sepsis and ICU patients [39–41]. Clinically, both the severity and duration of thrombocytopenia are associated with elevated mortality rates, whilst modest increases in platelet counts owing to secondary thrombocytosis are associated with improved patient outcomes [39–41]. Indeed, transfer of CD40L^hi^ platelets into early septic mice improved survival [59]; however, a better understanding of the mechanisms behind sepsis-induced thrombocytopenia is required given the heterogeneity in platelet abundance and coagulopathy between sepsis patients.

Our study is not without limitations. Gram-negative abdominal sepsis represents a fraction of the heterogeneous pathogens and sources of infection in clinical sepsis. Whilst *E. coli* are the most identified pathogens from sepsis patients, less frequent bacterial species can exhibit greater mortality in clinical sepsis. Indeed, a recent prospective cohort study of sepsis patients in Japan revealed that gram-positive methicillin-resistant *S. aureus* (MRSA) infection comprised approximately 3% of all cases; however, it displayed the greatest mortality rate at 48% [11]. Additionally, whilst diarrheal infections and appendicitis are prevalent underlying infectious causes of sepsis globally, respiratory and urinary tract infections also represent significant underlying causes of sepsis deaths [1, 2, 61]. Combined with tissue tropism displayed by key sepsis pathogens [11], this highlights the need for multiple preclinical models of sepsis that cover the breadth of common initiating pathogens and routes of infection. Indeed, the greater pulmonary or renal damage observed in clinical sepsis may be recapitulated using an intranasal or transurethral route of infection with alternate bacteria strains that display relevant tissue tropism. Our preclinical model provides a platform to establish such models, whereby alternate clinical bacterial species may be isolated and characterized, introduced via an appropriate route of infection, and titrated to the minimum infectious dose required for life-threatening organ dysfunction. Subsequently, the timing and nature of antibiotics comprising standard care will depend on the kinetics of organ dysfunction and pathogen utilized, respectively. Through this approach, new insights into the poorly characterized contribution of varied sepsis pathogens on multi-organ damage, independent of host inflammatory, physiological and metabolic dysfunction, may be revealed.

A further limitation of our experimental approach is attributed to a lack of heterogeneity in host factors. Multiple groups have reported sex differences in sepsis survival, with greater mortality rates observed in male patients and animals [2, 62–67]. Our study is limited by the exclusive use of male mice; thus, future mechanistic and therapeutic studies should utilize both male and female animals to identify sex-dimorphic effects. It is also important to acknowledge the contribution of pre-existing co-morbidities to sepsis mortality. Our gram-negative abdominal sepsis model represents a clinical best-case scenario: rapid identification of sepsis, appropriate antibiotic selection and no co-morbidities. Common co-morbidities observed in adult sepsis patients include diabetes, cancer, hypertension and chronic pulmonary, renal or cardiac disease [3, 11, 13, 61, 68]. The presence of such co-morbidities in sepsis increases mortality rates and likely contributes to the increase in sepsis mortality with age [1, 2, 68, 69]. Therefore, future studies should consider the optimal clinical care for septic aged mice in the context of common concurrent co-morbidities, whilst studies investigating mechanisms and therapeutics to address long-term morbidity in sepsis survivors may be better explored with young animals given the increased survival of patients between the extremities of age [1, 2, 68, 69]. Here, our model is advantageous over CLP methods as it lacks invasive surgery procedures to establish infection and minimizes experimental variability introduced by unknown infectious dosing. These are important considerations in aged mice experiments given their increased fragility, cost, and limited availability.

## Conclusions

Sepsis leads to dysregulation of multiple biological systems, yet current interventions for sepsis are confined to antimicrobials to eliminate the underlying infection and treatments to alleviate symptoms, with a lack of therapeutics aimed at limiting or preventing multi-organ failure. Our new preclinical model implements a standard clinical care regime at the onset of symptoms and recapitulates key disease outcomes observed in sepsis patients. Our model can be adapted with alternate clinical pathogens or routes of infection and applied to outbred mouse strains, mixed-sex and aged mice to establish multiple preclinical models that when combined, recapitulate the clinical heterogeneity necessary to identify and validate putative sepsis therapeutics. Additionally, our approach allows for the study of dysfunctional homeostasis in surviving animals, whereby mechanistic insights will reveal new targets for urgently needed therapeutic agents that improve the quality of life for surviving sepsis patients.

## Methods

### Animals

Male C57BL/6 J mice at 8–10 weeks of age were purchased from Monash Animal Research Platform and housed at the Monash Health Translation Precinct (MHTP) animal facility under specific pathogen-free conditions. Following transportation, the mice were acclimatized for a minimum period of 7 days before experimental investigation. Mice were housed in groups of less than five per cage in a 12 h light and 12 h dark cycle in a temperature-controlled environment (21 ± 3°C). Water and food pellets were provided to the mice *ad libitum*, and their cages were changed weekly.

### Bacteria strains and culturing

*E. coli* ST38 was isolated from the blood culture of a septic shock patient at Austin Health, Melbourne, Australia [14]. *E. coli* strain MER-172 was isolated from the bloodstream of a patient enrolled in the MERINO trial [22] and kindly provided by Prof Jeremy Barr, Monash University, Melbourne, Australia. *E. coli* HS is a commensal isolate previously obtained from a healthy individual [23]. *E. coli* HS and the *E. coli* lab strain DH5α were kindly provided by Prof Elizabeth Hartland, Hudson Institute, Melbourne, Australia. Frozen *E. coli* stocks were prepared in Luria Bertani (LB) broth (Oxoid) with 20% glycerol and stored at -80°C. For infection, frozen stocks were grown overnight in LB broth for 14 h at 37°C with 120 rpm agitation, then sub-cultured until mid-log growth was achieved, as predetermined by growth rate assays. Sub-cultures were washed with 0.9% saline, pelleted at 3000 g, then resuspended in saline for optical density measurements at 600 nm with a Tecan Infinite ELISA plate reader. Bacteria concentration (CFU/mL) was interpolated from individual standard curves for each strain, then bacteria were diluted in saline to 2×10^8^ CFU/mL for infection. Heat-killed *E. coli* were prepared by heating an aliquot of 2×10^8^ CFU/mL at 70°C for 15 min to achieve >99.99% sterilization. Bacteria concentration was validated by enumerating serial dilutions of samples plated on LB agar (Thermo) and incubated overnight at 37°C.

### Bacteria sequencing and bioinformatic analyses

For sequencing, *E. coli* ST38 was grown in Heart Infusion broth (Oxoid) at 37°C for 16 h. Genomic DNA was extracted from liquid cultures using the GenFind V3 Reagent Kit (Beckman Coulter) as per manufacturer’s instructions. Short read sequencing libraries were prepared using the Nextera Flex DNA Library Prep Kit (Illumina) and 150 bp paired-end sequencing performed on the NextSeq 500 System (Illumina). The genomic sequences of *E.* *coli* strains MER-172 and HS were accessed from the National Institute of Health Sequence Read Archive, SRS3773506 and SRR10997234, respectively. Bioinformatic analyses were performed with the online services hosted by the Center for Genomic Epidemiology (www.genomicepidemiology.org) using Illumina paired-end reads. Sequence type was determined using MLST2.0 version 2.0.9 [70]. In silico serotype identification was performed with SerotypeFinder 2.0 version 2.0.1 [71]. Virulence factors were identified with VirulenceFinder 2.0 version 2.0.3 [72, 73] and acquired antibiotic resistance genes were identified with ResFinder 4.1 [74]. Parameters for bioinformatic analyses were set as follows: 85% sequence identity for SerotypeFinder and VirulenceFinder, 90% sequence identity for ResFinder, and a minimum sequence coverage of 60% for all analyses. Phylogroup was determined via ClermonTyping version 21.03 [75] using a single genome FASTA sequence assembled by the online platform Pathogenwatch (Centre for Genomic Pathogen Surveillance).

### Sepsis model

Mice were injected intraperitoneally (i.p.) with 4×10^7^ CFU live or heat-killed *E. coli*, or saline. All infections were performed at 9AM to minimize circadian rhythm effects. Mice were monitored at regular intervals for clinical signs of disease, weight loss, and body temperature as measured by an infrared temperature gun (Famidoc). Clinical score evaluated mouse activity level, movement speed, alertness, weight loss, respiration, fur appearance and dehydration on a scale from 0 (normal) to 3 (severe) as outlined by the Monash University Animal Ethics Committee. Scoring was performed by one researcher to limit inter-researcher variability. For models with standard care, six doses of 50 μg/g Primaxin (MSD Australia), equivalent to 25 μg/g Imipenem and 25 μg/g Cilastatin, were administered in a total volume of 500 μL saline i.p. every 12 h, starting 12 h post-infection (Fig. 2A). Serial blood samples were collected from alternating saphenous veins 7 days prior to infection, then 12, 24, 48 and 78 h post-infection before the administration of antibiotics. Cohorts of mice were culled at timepoints delineating peak disease (12 h post-infection), end of standard care (78 h post-infection) and recovery (7 days post-infection), or earlier if predetermined humane endpoints were met.

### Organ harvest

Mice were deeply anaesthetized with isoflurane and blood was harvested from the inferior vena cava for bacterial burden enumeration, sera analyses and haematological analyses with a VetScan HM5 Haematology analyser (Abaxis). Organs were perfused in situ with 20 mL of phosphate buffered saline (PBS) via the left ventricle, then dissected, processed for histology and weighed for biological assays. Organ homogenates were prepared using Lysing Matrix S tubes (MP Biomedicals), 1 mL of sterile saline and processing with a Beadbug 6 (5 cycles at 4300 rpm for 30 s). Bacteria were enumerated from fresh blood or organ homogenates by serial dilution in saline, plating on LB agar and overnight incubation at 37°C. Organ homogenates and sera samples were subsequently stored at −80°C until use in biochemical and cytokine assays.

### Histology

Organ samples were fixed in 10% neutral buffered formalin for 24 h, then stored in 0.05% sodium azide/PBS, except kidneys which were stored in 80% ethanol (v/v). Paraffin embedding, sectioning at 3 μm (liver and kidney) or 4 μm (spleen), haematoxylin & eosin (H&E) staining or PAS staining, and slide scanning with an Aperio Scanscope AT Turbo at ×20 magnification was performed by the Monash Histology Platform. Images were viewed and analysed in Aperio ImageScope (v12.4.6.5003) and prepared for figures using Adobe Photoshop (v24.6.0). Histological features quantified in text were counted within 2 organ sections per mouse, then normalized to tissue area.

### Biochemical and cytokine assays

Sera was assayed for blood urea nitrogen (BUN) using the Urea Assay Kit (Abcam), and alanine transaminase (ALT) activity with the Alanine Aminotransferase Assay Kit (Merck) as per manufacturer’s instructions, then analysed on a Tecan Infinite ELISA plate reader. Cytokines were measured in sera and organ homogenates with the LEGENDplex Mouse Inflammation Panel (BioLegend) and LEGENDplex Mouse HSC Panel (BioLegend) as per manufacturer’s protocol, then acquired on a BD LSR-Fortessa X20 at the MHTP FlowCore.

### Statistics

All statistical comparisons were performed using GraphPad Prism v10.5.0 software. Brown–Forsythe and Bartlett’s tests were used to identify datasets with equal standard deviations, and tests for the normality of residues were used to identify nonparametric datasets. Statistical tests used are described in figure legends. Statistical significance in graphs is represented by *p < 0.05, **p < 0.01, ***p < 0.001 or **** < 0.0001.

## Supplementary Information

Additional file1 (DOCX 4198 KB)

## Data Availability

The datasets used and/or analysed during the current study are available from the corresponding author on reasonable request. *E. coli* ST38 genomic data are available from the National Institute of Health Sequence Read Archive, PRJNA1345804.
