# Supplementary material for: A new murine gram-negative sepsis model with standard care satisfies Sepsis-3 and reproduces clinical pathology
Source: Intensive Care Med Exp. 2026 Mar 30;14:39. doi: 10.1186/s40635-026-00886-5 (PMC13035989; doi:10.1186/s40635-026-00886-5)
Supplement: Supplementary file 1 — Additional file1 (DOCX 4198 KB) [file 40635_2026_886_MOESM1_ESM.docx]

**A new murine gram-negative sepsis model with standard care satisfies Sepsis-3 and reproduces clinical pathology**

Cameron R Bastow^1^, Cynthia Mei^1^, Shu Wen Wen^1^, Jenny L Wilson^1^, Huynh Nguyen^1^, Althea R Suthya^1^, Joshua H Bourne^1^, Yugeesh R Lankadeva^2,3,4^, Connie H Y Wong^1^

^1^ Centre for Inflammatory Diseases, Department of Medicine, School of Clinical Sciences at Monash Health, Monash University, Clayton, VIC, Australia

^2^ Translational Cardiovascular and Renal Research Group, Florey Institute of Neuroscience and Mental Health, The University of Melbourne, Melbourne, VIC, Australia

^3^ Department of Critical Care, Melbourne Medical School, The University of Melbourne, Melbourne, VIC, Australia

^4^ Department of Anaesthesia, Austin Hospital, Melbourne, VIC, Australia

**Supplemental Material:**

**Supplemental Figure 1:** Human *E coli* isolates utilized in this study have distinct phylogeny and virulence profiles

**Supplemental Figure 2:** Mice recover from sub-lethal infection with *E coli* ST38 without therapeutic intervention

**Supplemental Figure 3:** Concentration of serum inflammatory cytokines in untreated *E coli* sepsis mice after 12 hours

**Supplemental Figure 4:** Concentration of serum cytokines in *E coli* sepsis mice receiving standard care throughout recovery

**Supplemental Figure 5:** Concentration of splenic cytokines in *E coli* sepsis mice receiving standard care throughout recovery

**Supplemental Figure 6:** Concentration of hepatic cytokines in *E coli* sepsis mice receiving standard care throughout recovery

**Supplemental Figure 7:** Concentration of renal cytokines in *E coli* sepsis mice receiving standard care throughout recovery

**Supplemental Figure 8:** Concentration of pleural cytokines in *E coli* sepsis mice receiving standard care throughout recovery

**Supplemental Table 1.** Virulence factor genes identified in human *E coli* isolates

**Supplemental Table 2.** Antimicrobial resistance genes identified in human *E coli* isolates

**Supplemental Table 3.** Satisfaction of Minimal Quality Threshold in Preclinical Sepsis Studies (MQTiPSS) recommendations

**
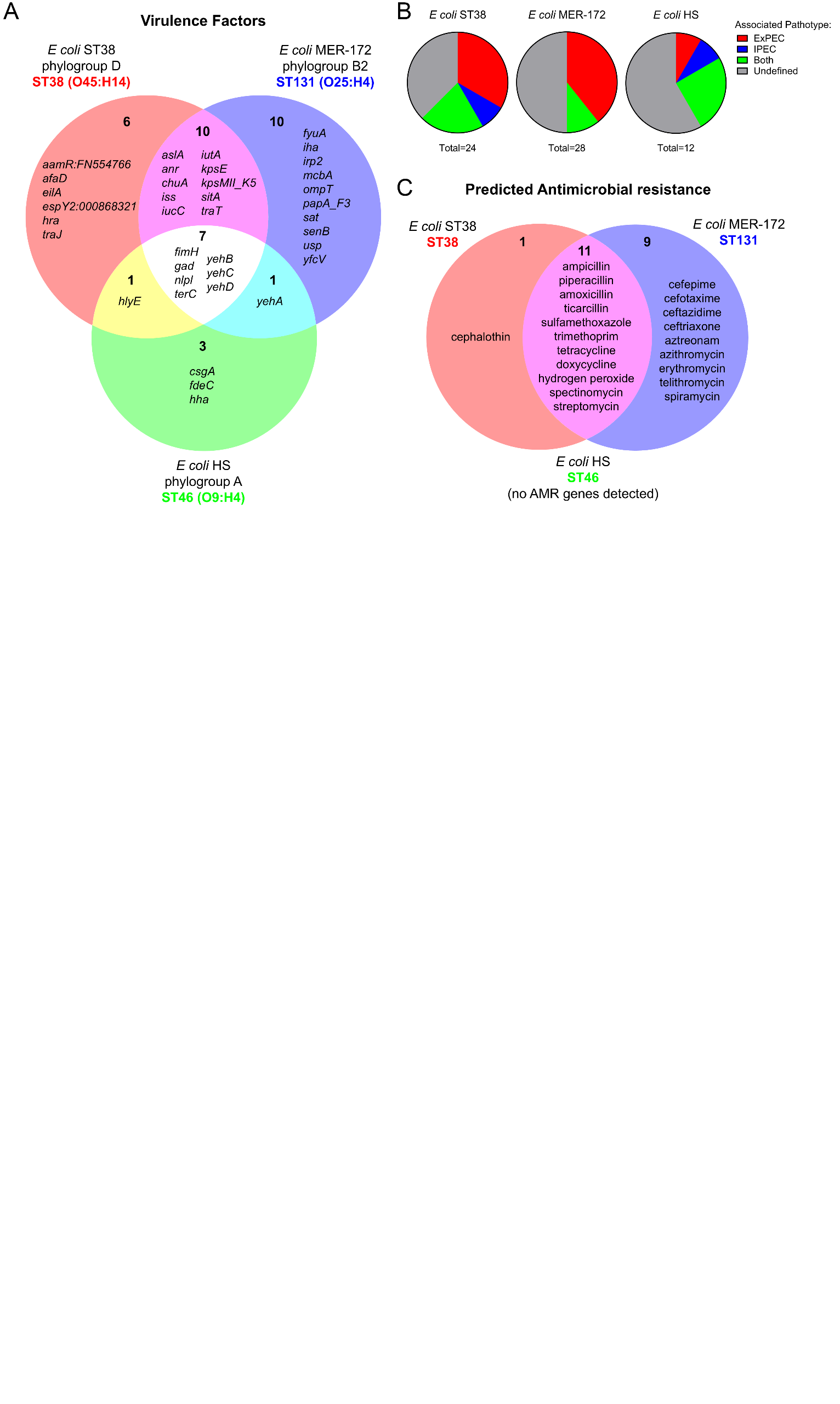
Supplemental Figure 1: Human *E coli* isolates utilized in this study have distinct phylogeny and virulence profiles**

Clinical *E coli* sepsis isolates utilized in this study include the clinical strains ST38 and MER‑172, and the commensal *E coli* HS strain. **A** Sequence type, phylogeny, serotype and virulence factor genes identified in *E coli* strains. Virulence factors are further described in Supplemental Table 1. **B** Total number of virulence factor genes and their associated *E coli* pathotype for each strain, ExPEC: extraintestinal pathogenic *E coli*, IPEC: intestinal pathogenic *E coli*. **C** Predicted antimicrobial resistances for each *E coli* strain. Antimicrobial resistant genes are described in Supplemental Table 2.

**
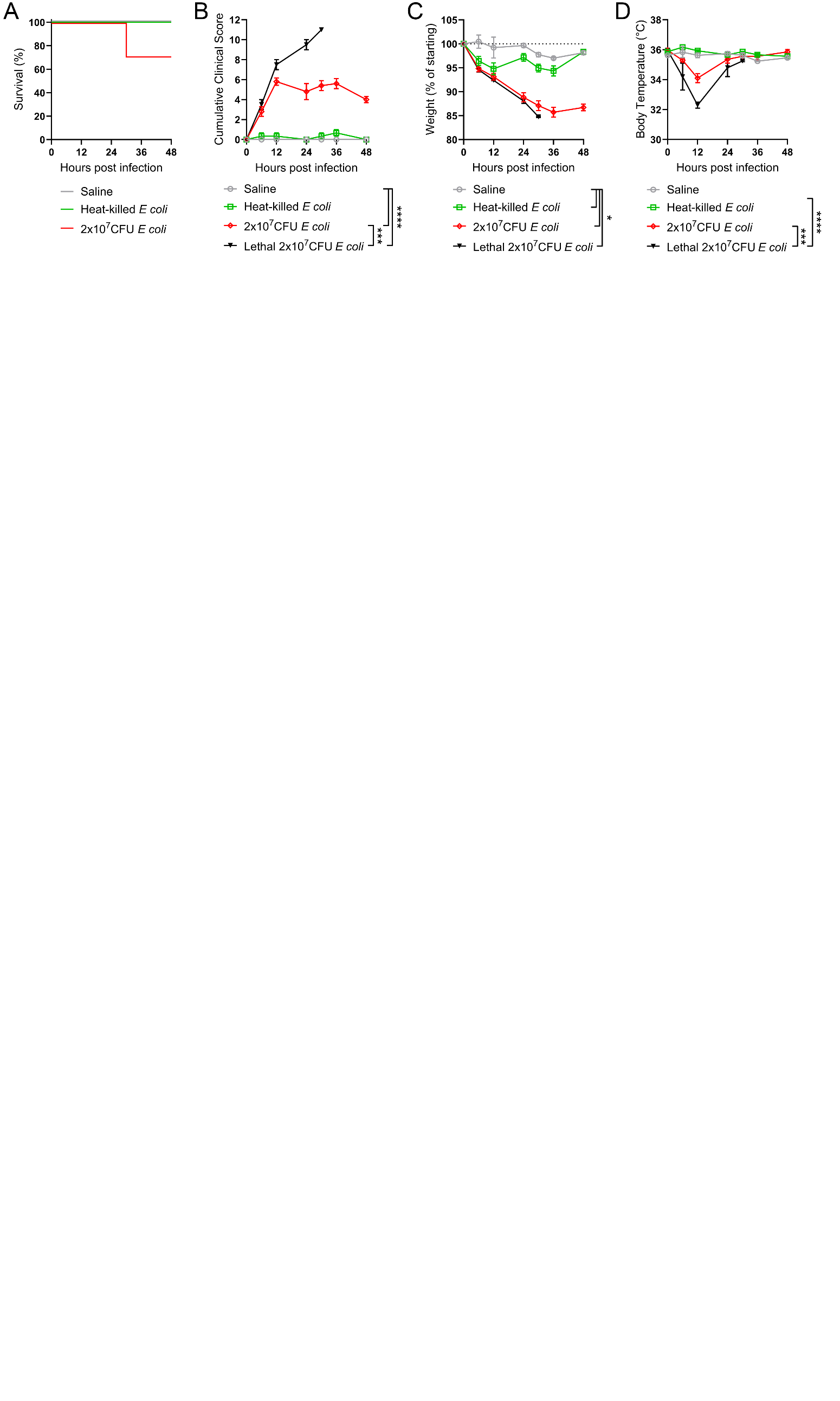
Supplemental Figure 2: Mice recover from sub-lethal infection with *E coli* ST38 without therapeutic intervention**

Mice were infected with 2×10^7^ CFU of *E coli* ST38 (half the lethal dose) and **A** survival, **B** cumulative clinical score, **C** weight loss and **D** body temperature were measured over the following 48 h. **A-D** Data pooled from 3 independent experiments, saline n=3, heat-killed *E coli* n=3, *E coli* ST38 n=7 mice. In (B, C, D) mice that succumbed to lethal infections (n=2) were separated from the 2×10^7^ CFU *E coli* group (n=5), displayed as mean ± SEM, two-way main effects ANOVA with Bonferroni’s multiple comparison test. *p<0.05, ***p<0.001, ****p<0.0001.

**
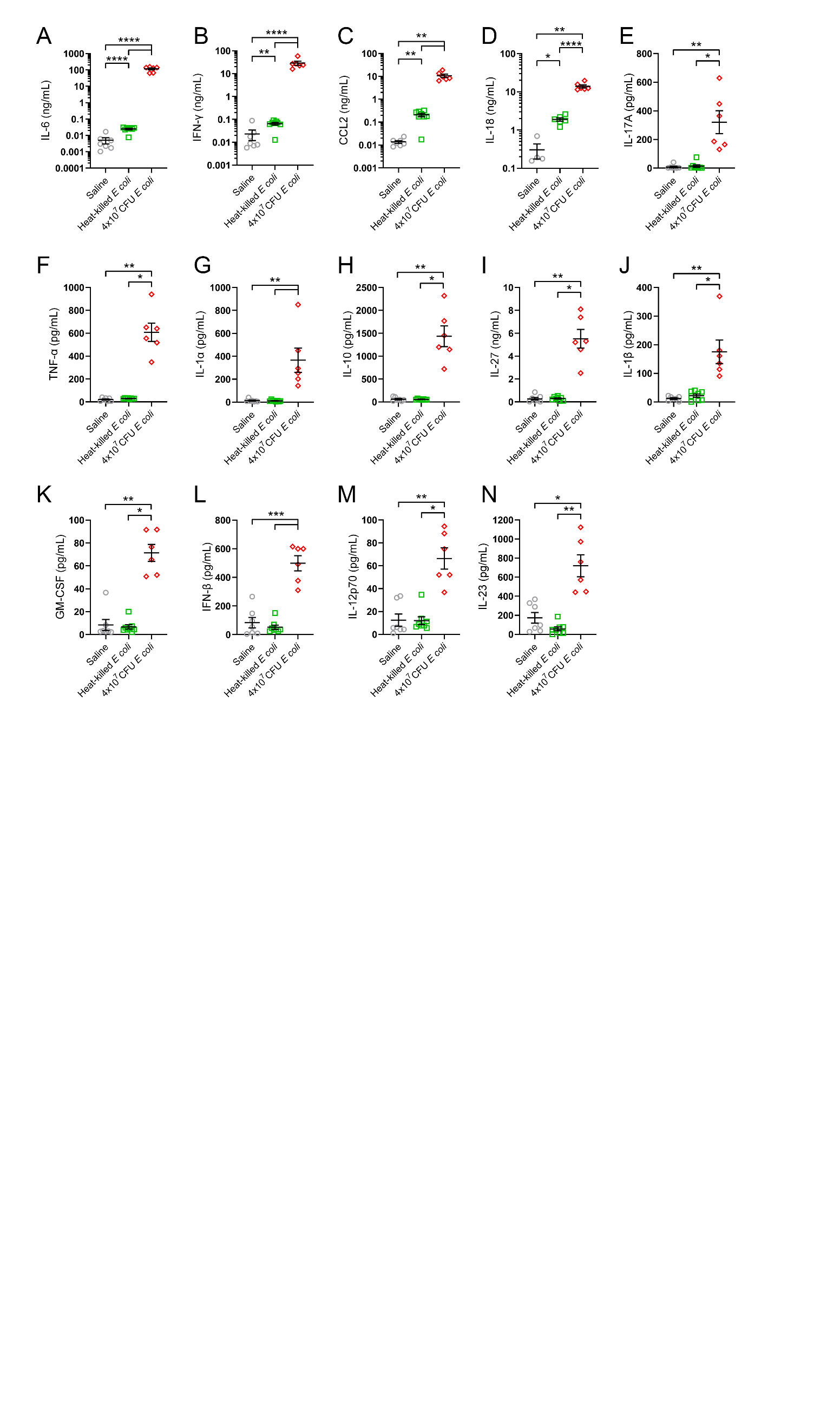
Supplemental Figure 3: Concentration of serum inflammatory cytokines in untreated *E coli* sepsis mice after 12 hours**

Mice were infected intraperitoneally (i.p.) with 4×10^7^ CFU of *E coli* ST38 and serum inflammatory cytokines **A‑N** were measured at 12 h post infection. See Figure 1H for log2-fold increases relative to saline control. Data pooled from 3 independent experiments, saline n=7 (except for IL-18, n=4), heat-killed *E coli* n=8, *E coli* ST38 n=6 mice/analyte, each symbol is one mouse with mean ± SEM. **A, B** Lognormal ordinary ANOVA with Tukey’s multiple comparisons test, **C, L, N** Brown-Forsythe and Welch ANOVA with Dunnet’s T3 multiple comparisons test, **D** Lognormal Brown-Forsythe and Welch ANOVA with Dunnet’s T3 multiple comparisons test, **E-K, M** Kruskal Wallis ANOVA with Dunn’s multiple comparisons test. *p<0.05, **p<0.01, ***p<0.001, ****p<0.0001.

**
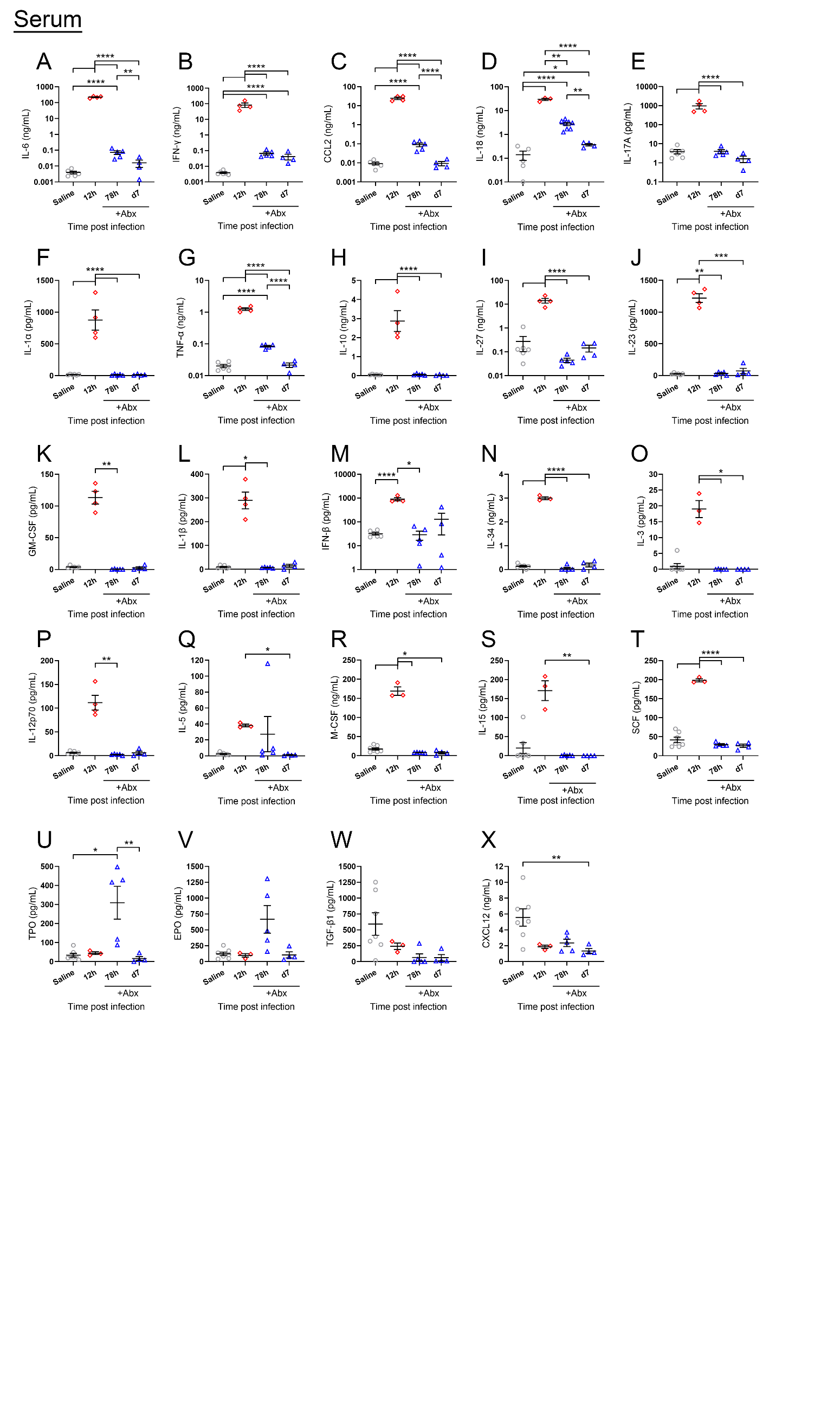
Supplemental Figure 4: Concentration of serum cytokines in *E coli* sepsis mice receiving standard care throughout recovery**

Mice were infected i.p. with 4×10^7^ CFU of *E coli* ST38 and treated with antibiotics and fluid resuscitation. Serum inflammatory and haematopoietic cytokines **A-X** were measured at 12 h, 78 h and 7 days post infection, and in healthy saline control mice. See Figure 3A for log2-fold increases relative to saline control. Data pooled from 5 independent experiments, saline n=6 **A-C, E-M, P**, n=5 **D**, n=7 **N, O, Q-X**; 12 h n=4 **A-C, E-M, P**, n=3 **D, N, O, Q-X**; 78 h n=5 except **D**, n=8; d7 n=4 mice/analyte, each symbol is one mouse with mean ± SEM. **A-I, X** Lognormal ordinary ANOVA with Tukey’s multiple comparisons test, **J, R, W** Brown-Forsythe and Welch ANOVA with Dunnet’s T3 multiple comparisons test, **K, L, O, P, Q, S, U, V** Kruskal Wallis ANOVA with Dunn’s multiple comparisons test, **M** Lognormal Brown-Forsythe and Welch ANOVA with Dunnet’s T3 multiple comparisons test, **N, T** Ordinary one-way ANOVA with Tukey’s multiple comparison test. *p<0.05, **p<0.01, ***p<0.001, ****p<0.0001.

**
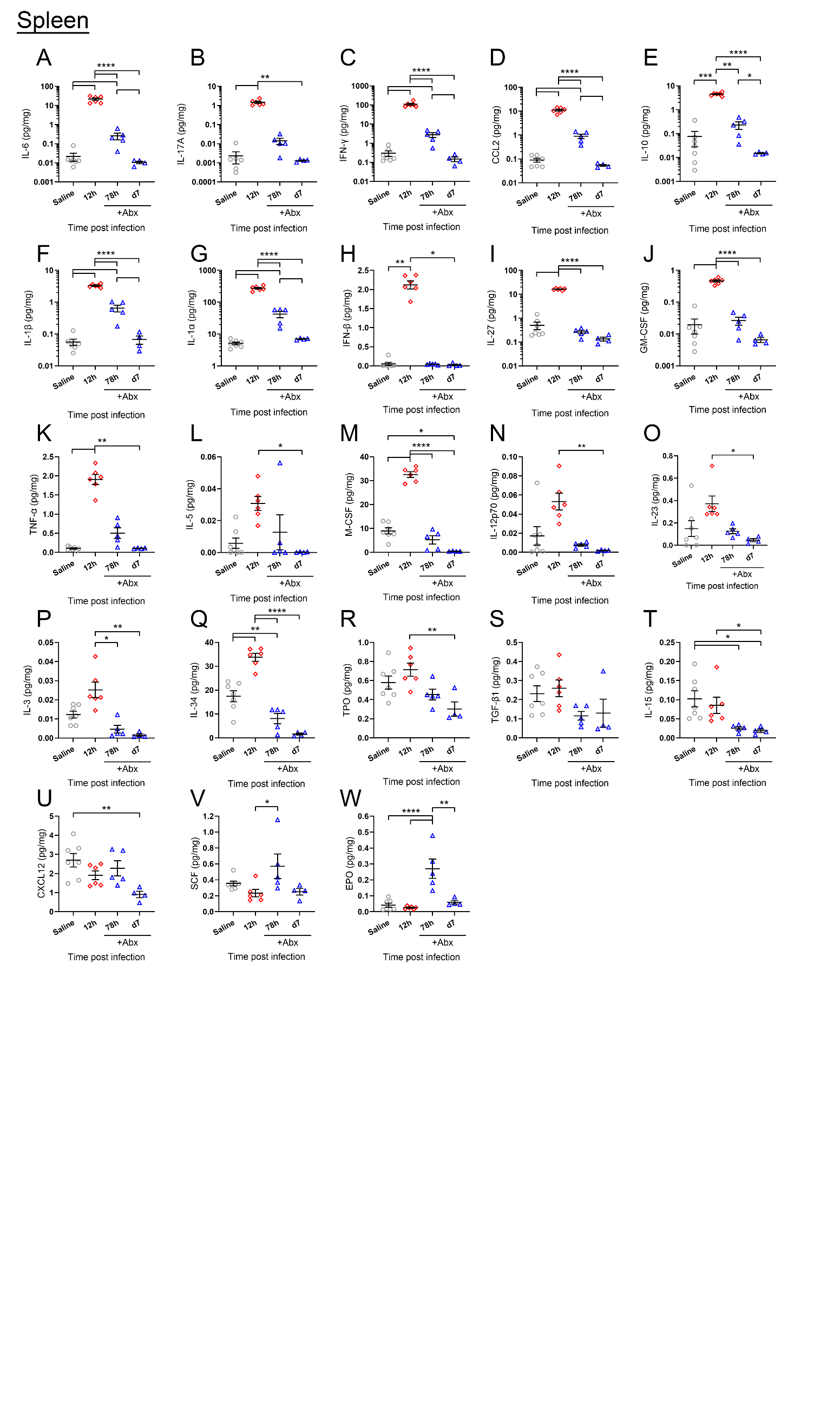
Supplemental Figure 5: Concentration of splenic cytokines in *E coli* sepsis mice receiving standard care throughout recovery**

Mice were infected i.p. with 4×10^7^ CFU of *E coli* ST38 and treated with antibiotics and fluid resuscitation. Inflammatory and haematopoietic cytokines **A-W** were measured in spleen homogenates at 12 h, 78 h and 7 days post infection, and in healthy saline control mice. See Figure 3B for log2-fold increases relative to saline control. Data pooled from 5 independent experiments, saline n=7, 12 h n=6, 78 h n=5, d7 n=4 mice/analyte, each symbol is one mouse with mean ± SEM. **A, C, D, F, G, J, W** Lognormal ordinary ANOVA with Tukey’s multiple comparisons test, **B, H, K, L, N, O, P, T, V** Kruskal Wallis ANOVA with Dunn’s multiple comparisons test, **E, I** Lognormal Brown-Forsythe and Welch ANOVA with Dunnet’s T3 multiple comparisons test, **M, Q** Brown-Forsythe and Welch ANOVA with Dunnet’s T3 multiple comparisons test, **R, S, U** Ordinary one-way ANOVA with Tukey’s multiple comparison test. . *p<0.05, **p<0.01, ***p<0.001, ****p<0.0001.

**
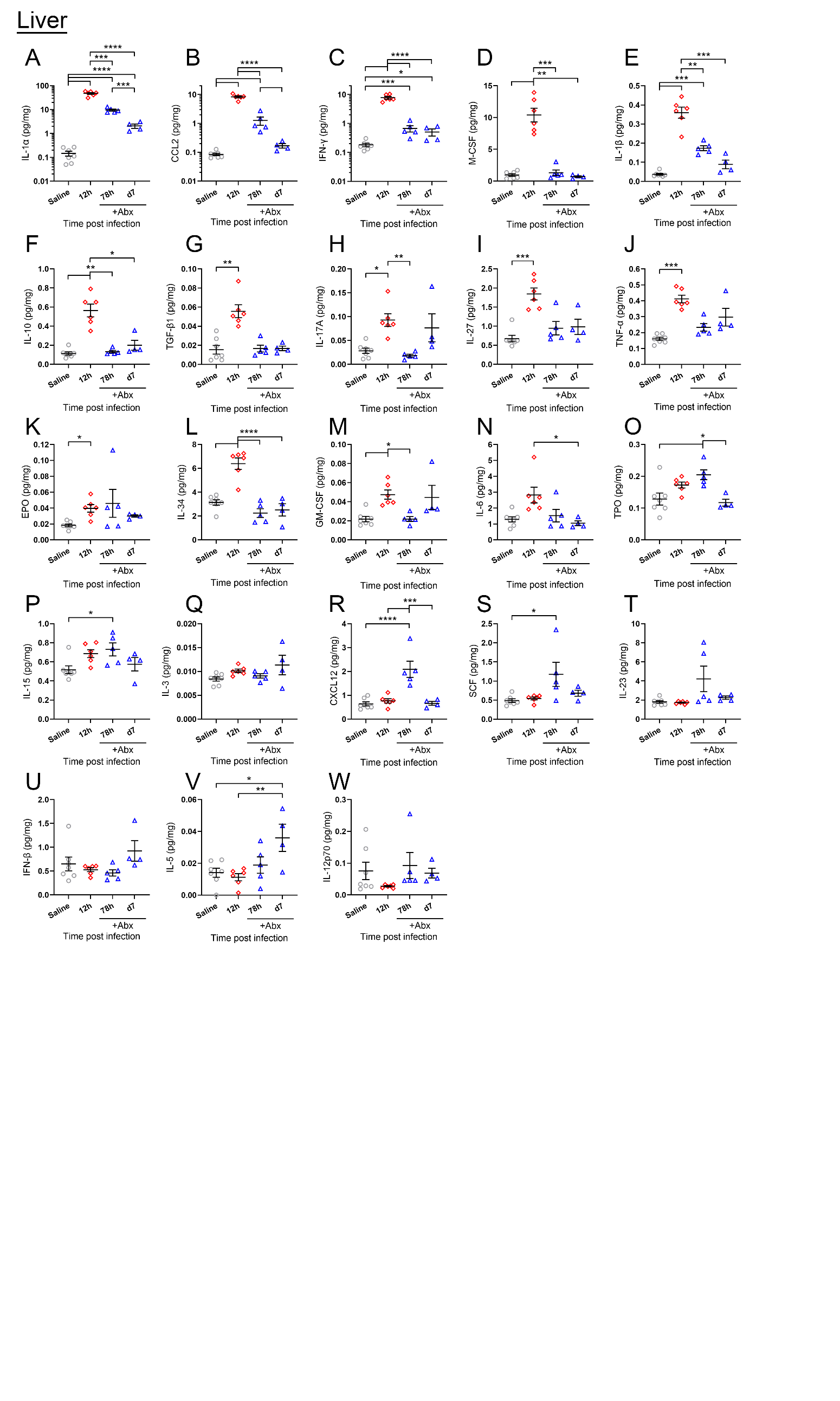
Supplemental Figure 6: Concentration of hepatic cytokines in *E coli* sepsis mice receiving standard care throughout recovery**

Mice were infected i.p. with 4×10^7^ CFU of *E coli* ST38 and treated with antibiotics and fluid resuscitation. Inflammatory and haematopoietic cytokines **A-W** were measured in liver homogenates at 12 h, 78 h and 7 days post infection, and in healthy saline control mice. See Figure 3C for log2-fold increases relative to saline control. Data pooled from 5 independent experiments, saline n=7, 12 h n=6, 78 h n=5, d7 n=4 mice/analyte, each symbol is one mouse with mean ± SEM. **A, B, C, R** Lognormal ordinary ANOVA with Tukey’s multiple comparisons test, **D, E, F** Brown-Forsythe and Welch ANOVA with Dunnet’s T3 multiple comparisons test, **G-K, M, N, O, Q, S, T, U, W** Kruskal Wallis ANOVA with Dunn’s multiple comparisons test, **L, P, V** Ordinary one-way ANOVA with Tukey’s multiple comparison test. *p<0.05, **p<0.01, ***p<0.001, ****p<0.0001.

**
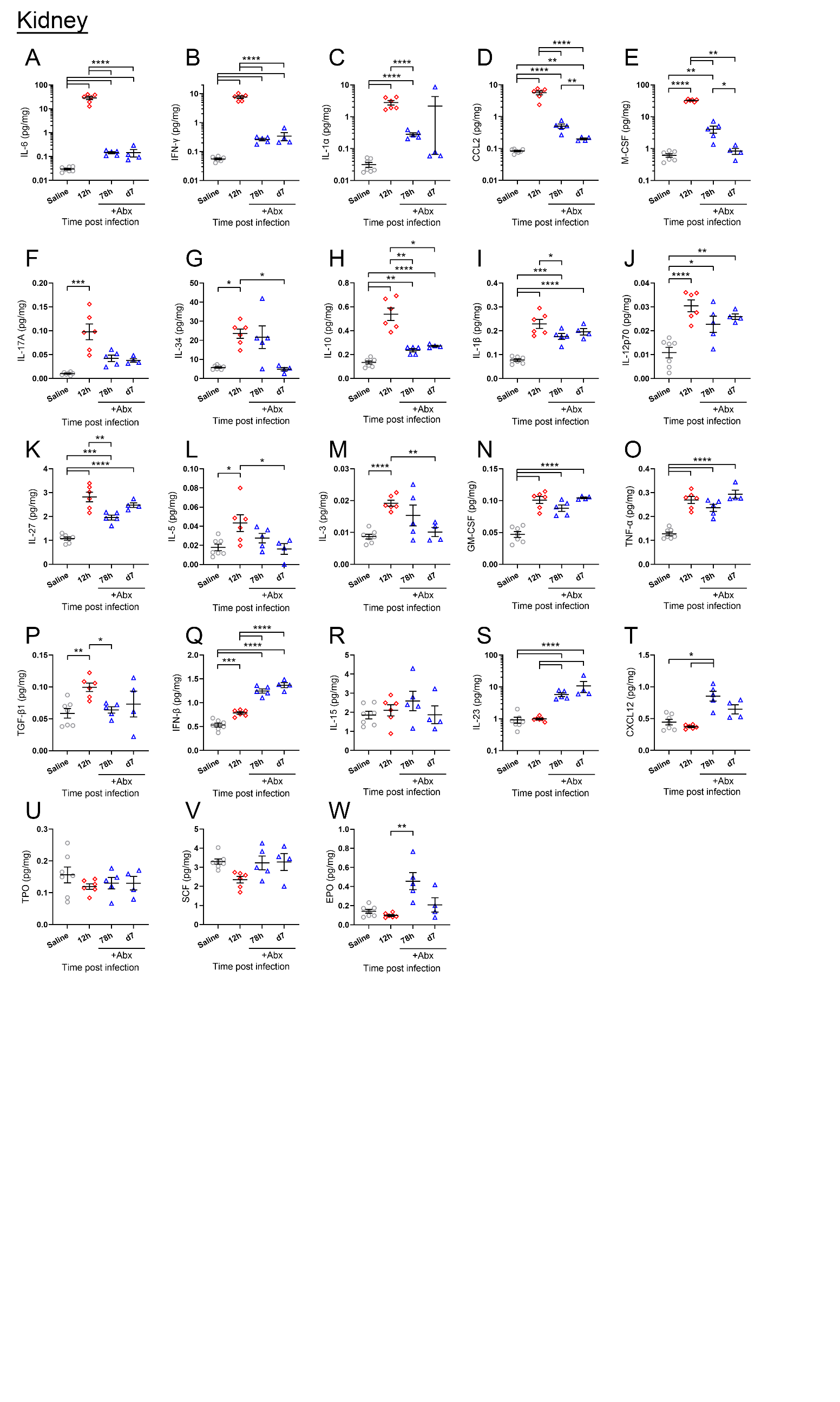
Supplemental Figure 7: Concentration of renal cytokines in *E coli* sepsis mice receiving standard care throughout recovery**

Mice were infected i.p. with 4×10^7^ CFU of *E coli* ST38 and treated with antibiotics and fluid resuscitation. Inflammatory and haematopoietic cytokines **A-W** were measured in kidney homogenates at 12 h, 78 h and 7 days post infection, and in healthy saline control mice. See Figure 3D for log2-fold increases relative to saline control. Data pooled from 5 independent experiments, saline n=7, 12 h n=6, 78 h n=5, d7 n=4 mice/analyte, each symbol is one mouse with mean ± SEM. **A, B, D, S** Lognormal ordinary ANOVA with Tukey’s multiple comparisons test, **C, E** Lognormal Brown-Forsythe and Welch ANOVA with Dunnet’s T3 multiple comparisons test, **F, G, W** Kruskal Wallis ANOVA with Dunn’s multiple comparisons test, **H, M, P, T** Brown-Forsythe and Welch ANOVA with Dunnet’s T3 multiple comparisons test, **I-L, N, O, Q, R, U, V** Ordinary one-way ANOVA with Tukey’s multiple comparison test. *p<0.05, **p<0.01, ***p<0.001, ****p<0.0001.

**
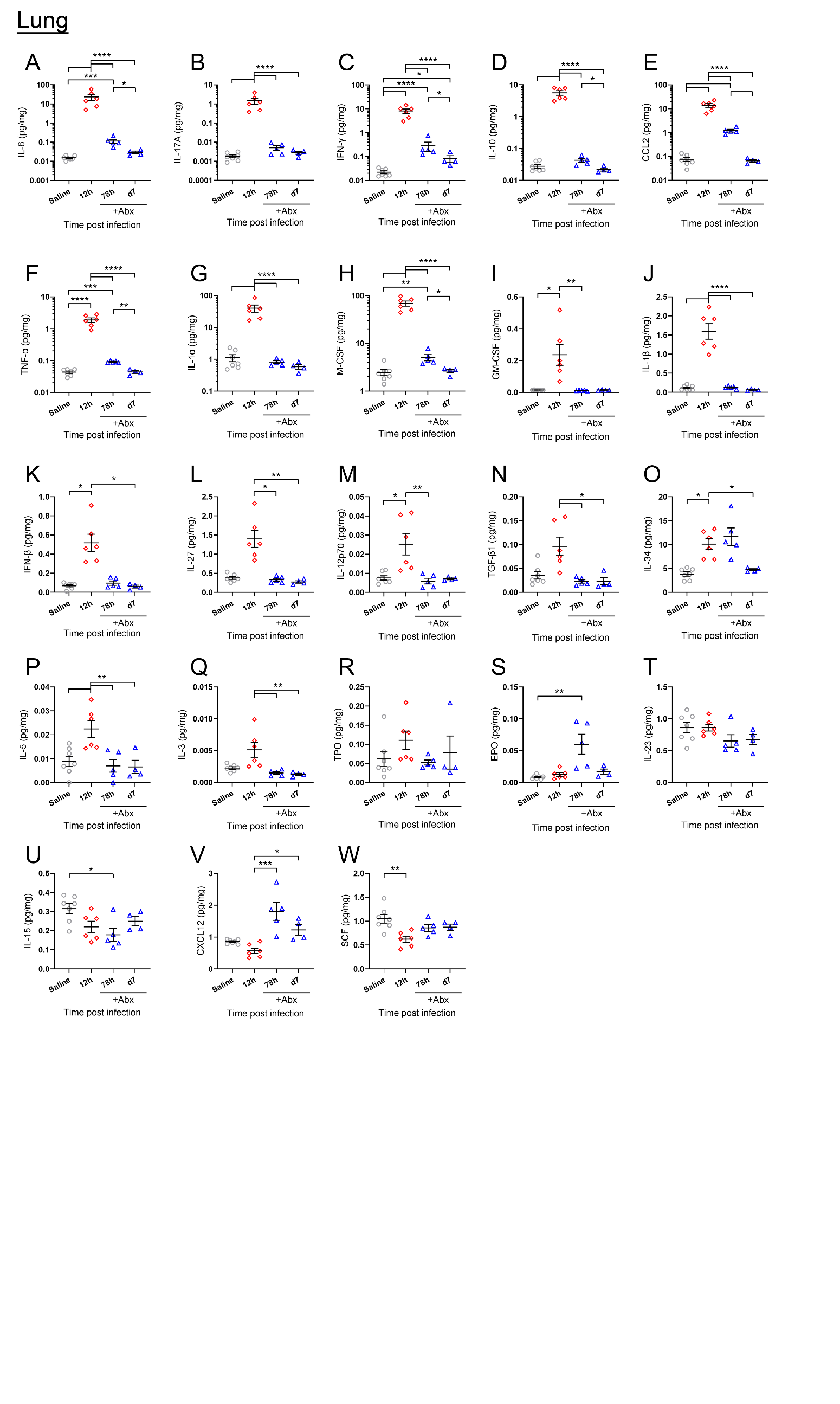
Supplemental Figure 8: Concentration of pleural cytokines in *E coli* sepsis mice receiving standard care throughout recovery**

Mice were infected i.p. with 4×10^7^ CFU of *E coli* ST38 and treated with antibiotics and fluid resuscitation. Inflammatory and haematopoietic cytokines **A-W** were measured in lung homogenates at 12 h, 78 h and 7 days post infection, and in healthy saline control mice. See Figure 3E for log2-fold increases relative to saline control. Data pooled from 5 independent experiments, saline n=7, 12 h n=6, 78 h n=5, d7 n=4 mice/analyte, each symbol is one mouse with mean ± SEM. **A-H, J** Lognormal ordinary ANOVA with Tukey’s multiple comparisons test, **I, K-N, Q, R, S, V** Kruskal Wallis ANOVA with Dunn’s multiple comparisons test, **O** Brown-Forsythe and Welch ANOVA with Dunnet’s T3 multiple comparisons test, **P, T, U, W** Ordinary one-way ANOVA with Tukey’s multiple comparison test. *p<0.05, **p<0.01, ***p<0.001, ****p<0.0001.

**Supplemental Table 1. Virulence factor genes identified in human *E coli* isolates**

| Strain | Virulence gene | Identity/Function | Associated pathotype |
| --- | --- | --- | --- |
| ST38, MER-172 | *aslA* | Putative sulfatase | ND |
| ST38 | *aamR:FN554766* | Unknown | ND |
| ST38 | *afaD* | Afimbrial adhesion | IPEC (DAEC) / ExPEC (UPEC) [1, 2] |
| ST38, MER-172 | *anr* | AraC negative regulator |  |
| ST38, MER-172 | *chuA* | Outer membrane hemin receptor | ExPEC (UPEC, SEPEC) [2] |
| HS | *csgA* | Curlin major subunit | IPEC (EPEC, EHEC) [1] |
| ST38 | *eilA* | Salmonella HilA homolog | IPEC (EAEC) [3] |
| ST38 | *espY2:000868321* | non-LEE-encoded type III secreted effector from *E coli* O157:H7 strain TW14359 | IPEC (EAEC) [4] |
| HS | *fdeC* | intimin-like adhesin FdeC | IPEC/ExPEC [5] |
| ST38, HS, MER-172 | *fimH* | Type 1 fimbriae | IPEC/ExPEC [1, 2] |
| MER-172 | *fyuA* | Siderophore receptor | ND |
| ST38, HS, MER-172 | *gad* | Glutamate decarboxylase | ND |
| HS | *hha* | Haemolysin expression modulator Hha (previously rmoA) | ND |
| ST38, HS | *hlyE* | Avian *E coli* haemolysin | IPEC (EAEC) / ExPEC (APEC) [1, 2] |
| ST38 | *hra* | Heat-resistant agglutinin | ExPEC [6] |
| MER-172 | *iha* | Adherence protein | ExPEC (UPEC) [2] |
| MER-172 | *irp2* | High molecular weight protein 2 non-ribosomal peptide synthetase | ND |
| ST38, MER-172 | *iss* | Increased serum survival | ExPEC (NMEC, SEPEC, APEC) [2] |
| ST38, MER-172 | *iucC* | Aerobactin synthetase | ExPEC (APEC, UPEC) [2] |
| ST38, MER-172 | *iutA* | Ferric aerobactin receptor | IPEC (AIEC) / ExPEC (APEC, NMEC, UPEC) [1, 2] |
| ST38, MER-172 | *kpsE* | Capsule polysaccharide export inner-membrane protein | ExPEC [6] |
| ST38, MER-172 | *kpsMII_K5* | Polysialic acid transport protein; Group 2 capsule | IPEC (AIEC) / ExPEC (NMEC, SEPEC) [1, 2] |
| MER-172 | *mcbA* | Bacteriocin microcin B17 | ND |
| ST38, HS, MER-172 | *nlpl* | Lipoprotein nlpl precursor | ND |
| MER-172 | *ompT* | Outer membrane protease (protein protease 7) | ExPEC (NMEC, UPEC) [2] |
| MER-172 | *papA_F43* | Major pilin subunit F43 | ExPEC (UPEC, SEPEC, APEC) [2] |
| MER-172 | *sat* | Serine protease autotransporters of Enterobacteriaceae (SPATE) | ExPEC (UPEC) [2] |
| MER-172 | *senB* | Plasmic-encoded enterotoxin | ND |
| ST38, MER-172 | *sitA* | Iron transport protein | ExPEC (APEC, UPEC) [2] |
| ST38, HS, MER-172 | *terC* | Tellurium ion resistance protein | ExPEC [6] |
| ST38 | *traJ* | Protein TraJ | ND |
| ST38, MER-172 | *traT* | Outer membrane protein complement resistance | ExPEC (NMEC, SEPEC, APEC) [2] |
| MER-172 | *usp* | Uropathogenic specific protein | ND |
| MER-172, HS | *yehA* | Outer membrane lipoprotein, YHD fimbriael cluster | ND |
| ST38, HS, MER-172 | *yehB* | Usher YHD fimbriael cluster | ND |
| ST38, HS, MER-172 | *yehC* | Chaperone YHD fimbriael cluster | ND |
| ST38, HS, MER-172 | *yehD* | Major pilin subunit, YHD fimbriael cluster | ND |
| MER-172 | *yfcV* | Fimbrial protein | ND |

AIEC, adherent-invasive *E coli*. APEC, avian pathogenic *E coli*. DAEC, diffusely adherent *E coli*. EAEC, enteroaggregative *E coli*. EHEC, enterohemorrhagic *E coli*. EPEC, enteropathogenic *E coli*. ExPEC, extraintestinal pathogenic *E coli*. IPEC, intestinal pathogenic *E coli*. NMEC, neonatal meningitis *E coli*. SEPEC, sepsis-associated *E coli*. UPEC, uropathogenic *E coli*. ND, not determined.

**Supplemental Table 2. Antimicrobial resistance genes identified in human *E coli* isolates**

| Strain | Resistance gene(s) | Antimicrobial class |
| --- | --- | --- |
| ST38 | *aadA1* | Aminocyclitol, Aminoglycoside |
| MER-172 | *aadA5* | Aminocyclitol, Aminoglycoside |
| ST38, MER-172 | *aph(3”)-Ib*; *aph(6)-Id* | Aminoglycoside |
| MER-172 | *blaCTX-M-27* | Beta-lactam |
| ST38 | *blaTEM-1C* | Beta-lactam |
| ST38 | *dfrA1* | Folate pathway antagonist |
| MER-172 | *dfrA17* | Folate pathway antagonist |
| MER-172 | *mph(A)* | Macrolide |
| ST38, MER-172 | *sitABCD* | Peroxide |
| ST38, MER-172 | *sul1*; *sul2* | Folate pathway antagonist |
| ST38, MER-172 | *tet(A)* | Tetracycline |

**Supplemental Table 3. Satisfaction of Minimal Quality Threshold in Preclinical Sepsis Studies (MQTiPSS) recommendations**

| MQTiPSS Recommendations and *Considerations* [7] | Achieved (Y/N) | Explanation |
| --- | --- | --- |
| Study Design   1. Survival follow-up should reflect the clinical time course of the sepsis model | Y | Mice were followed up to 7 days post infection |
| 1. Therapeutic interventions should be initiated after the septic insult replicating clinical care | Y | Antibiotics and fluid resuscitation were administered 12 h post infection |
| 1. Treatment should be randomized and blinded when feasible | N/A | No different treatments were given between experimental groups |
| 1. Provide as much information as possible (e.g. ARRIVE guidelines) on the model and methodology to enable replication | Y | All requirements of ARRIVE guidelines present within manuscript and/or available on request from corresponding author |
| - 1. *Replication of the findings in models that include co-morbidity and/or other biological variables (i.e. age, gender, diabetes, cancer, immuno-suppression, genetic background and others)* | N | Study only utilized adult male C57BL6J mice. Current studies are optimizing this model for aged mice (>18 months) with existing comorbidities |
| - 1. *In addition to rodents (mice and rats), consider modelling sepsis also in other (mammal) species* | Y | The same *E coli* ST38 strain is utilized in ovine models of sepsis within the Lankadeva Laboratory [8] |
| - 1. *Consider need for source control* | Y | Systemic imipenem/cilastatin antibiotic administration clears blood and organ bacterial burden |
| Human Modelling   1. The development and validation of standardized criteria to monitor the well-being of septic animals | Y | Weight, body temperature and clinical signs of illness are measured up to three times daily and scored as per criteria established and approved by the Monash Medical Centre Animal Ethics Committee |
| 1. The development and validation of standardized euthanasia of septic animals is recommended (exceptions possible) | Y | Humane endpoint criteria for sepsis mice have been established and approved by the Monash Medical Centre Animal Ethics Committee |
| 1. Analgesics recommended for surgical sepsis consistent with ethical considerations | N/A | No surgical procedures are performed in this sepsis model |
| 1. *Consider analgesics for nonsurgical sepsis* | N | Analgesics were not administered |
| Infection Types   1. We recommend that challenge with LPS is not an appropriate model for replicating human sepsis | Y | Sepsis model utilized live bacteria |
| 1. We recommend that microorganisms used in animal models preferentially replicate those commonly found in human sepsis | Y | *E coli* strains ST38 and MER-172 were isolated from the blood of sepsis patients |
| 1. *Consider modelling sepsis syndromes that are initiated at sites other than the peritoneal cavity (e.g., lung, urinary tract, brain)* | N | A common route of infection for ExPEC *E coli* strains is translocation across the gut, thus abdominal infection was utilized |
| Organ Failure/Dysfunction   1. Organ/system dysfunction is defined as life-threatening deviation from normal for that organ/system based on objective evidence | Y | Liver and kidney dysfunction, as measured by blood ALT and BUN, were significantly elevated in septic mice compared to healthy controls. Without intervention, this infection was 100% lethal |
| 1. Not all activities in an individual organ/system need to be abnormal for organ dysfunction to be present | Y | Single biochemical measures of organ dysfunction were utilized given the limited volumes of blood that could ethically be collected from mice with repeated sampling methods. Histological scoring supported these measures |
| 1. To define objective evidence of the severity of organ/system dysfunction, a scoring system should be developed, validated, and used or use an existing scoring system | Y | Levels of ALT and BUN were significantly elevated in septic mice compared to healthy controls |
| 1. Not all experiments must measure all parameters of organ dysfunction but animal models should be fully exploited | Y | ALT and BUN were measured in all experiments with repeated blood sampling |
| 1. *Avoid hypoglycemia* | N/A | Glucose levels were not monitored in this study |
| Fluid Resuscitation   1. Fluid resuscitation is essential unless part of the study | Y | Fluid resuscitation was administered |
| 1. Administer fluid resuscitation based on the specific requirements of the model | Y | Fluid resuscitation was paired with antibiotics administration at 12 h post infection when signs of organ dysfunction were evident |
| 1. Consider the specific sepsis model for the timing of the start and continuation for fluid resuscitation | Y | Fluid resuscitation initiated at 12 h post infection when signs of organ dysfunction were evident and maintained until recovery |
| 1. Resuscitation with iso-osmolar crystalloid solutions is recommended | Y | Fluid resuscitation performed with sterile 0.9% saline solution |
| 1. *Consider using pre-defined endpoints for fluid resuscitation, as necessary* | Y | 6 doses of fluid resuscitation every 12 h was used |
| 1. *Avoid fluid overload* | Y | Mice were not monitored for fluid overload, however the 500μL bolus of fluids i.p. is below the recommended maximum fluid bolus of 50% blood volume (~6% body weight) for mice weighing 25g. |
|  |  |  |
| Antimicrobial Therapy   1. Antimicrobials are recommended for pre-clinical studies assessing potential human therapeutics | Y | Antimicrobials were administered |
| 1. Antimicrobials should be chosen based on the model and likely/known pathogen | Y | Imipenem/cilastatin is a broad-spectrum carbapenem antibiotic used for the treatment of bacterial sepsis. The *E coli* ST38 strain utilized in this study is susceptible to imipenem/cilastatin |
| 1. Administration of antimicrobials should mimic clinical practice | Y | Antibiotic treatment initiated at 12 h post infection when signs of organ dysfunction in are first evident. This timepoint was chosen to best represent initiation of antibiotics of clinical sepsis. Sustained intravenous delivery of antibiotics in mice is technically-challenging and requires surgery, therefore antibiotics were delivered i.p. |
| 1. *Antimicrobials should be initiated after sepsis is established* | Y | Antibiotics treatment initiated 12 h post infection |

**Supplemental Material References**

1. Pakbin B, Brück WM, Rossen JWA (2021) Virulence Factors of Enteric Pathogenic Escherichia coli: A Review. Int J Mol Sci 22:9922. https://doi.org/10.3390/ijms22189922

2. Sarowska J, Futoma-Koloch B, Jama-Kmiecik A, et al (2019) Virulence factors, prevalence and potential transmission of extraintestinal pathogenic Escherichia coli isolated from different sources: recent reports. Gut Pathogens 11:10. https://doi.org/10.1186/s13099-019-0290-0

3. Sheikh J, Dudley EG, Sui B, et al (2006) EilA, a HilA-like regulator in enteroaggregative Escherichia coli. Molecular Microbiology 61:338–350. https://doi.org/10.1111/j.1365-2958.2006.05234.x

4. Boisen N, Scheutz F, Rasko DA, et al (2012) Genomic Characterization of Enteroaggregative Escherichia coli From Children in Mali. The Journal of Infectious Diseases 205:431–444. https://doi.org/10.1093/infdis/jir757

5. Nesta B, Spraggon G, Alteri C, et al (2012) FdeC, a Novel Broadly Conserved Escherichia coli Adhesin Eliciting Protection against Urinary Tract Infections. mBio 3:e00010-12. https://doi.org/10.1128/mBio.00010-12

6. Malberg Tetzschner AM, Johnson JR, Johnston BD, et al (2020) In Silico Genotyping of Escherichia coli Isolates for Extraintestinal Virulence Genes by Use of Whole-Genome Sequencing Data. J Clin Microbiol 58:e01269-20. https://doi.org/10.1128/JCM.01269-20

7. Osuchowski MF, Ayala A, Bahrami S, et al (2018) Minimum quality threshold in pre-clinical sepsis studies (MQTiPSS): an international expert consensus initiative for improvement of animal modeling in sepsis. Intensive Care Med Exp 6:26. https://doi.org/10.1186/s40635-018-0189-y

8. Lankadeva YR, Kosaka J, Evans RG, May CN (2018) An Ovine Model for Studying the Pathophysiology of Septic Acute Kidney Injury. In: Tharakan B (ed) Traumatic and Ischemic Injury: Methods and Protocols. Springer, New York, NY, pp 207–218
